# Vaginal microbiota molecular profiling and diagnostic performance of artificial intelligence-assisted multiplex PCR testing in women with bacterial vaginosis: a single-center experience

**DOI:** 10.3389/fcimb.2024.1377225

**Published:** 2024-04-05

**Authors:** Sihai Lu, Zhuo Li, Xinyue Chen, Fengshuangze Chen, Hao Yao, Xuena Sun, Yimin Cheng, Liehong Wang, Penggao Dai

**Affiliations:** ^1^ National Engineering Research Center for Miniaturized Detection Systems, Northwest University, Xi’an, China; ^2^ Department of Research and Development, Shaanxi Lifegen Co., Ltd., Xi’an, China; ^3^ Clinical Laboratory, The First Affiliated Hospital of Xi’an Medical University, Xi’an, China; ^4^ Academic Center, Henry M Gunn High School, Palo Alto, CA, United States; ^5^ Department of Obstetrics and Gynecology, The Hospital of Xi’ an Shiyou University, Xi’an, China; ^6^ Department of Obstetrics and Gynecology, Qinghai Red Cross Hospital, Qinghai, Xining, China

**Keywords:** BV diagnosis, machine learning, mPCR, *Lactobacillus* spp., CST

## Abstract

**Background:**

Bacterial vaginosis (BV) is a most common microbiological syndrome. The use of molecular methods, such as multiplex real-time PCR (mPCR) and next-generation sequencing, has revolutionized our understanding of microbial communities. Here, we aimed to use a novel multiplex PCR test to evaluate the microbial composition and dominant lactobacilli in non-pregnant women with BV, and combined with machine learning algorithms to determine its diagnostic significance.

**Methods:**

Residual material of 288 samples of vaginal secretions derived from the vagina from healthy women and BV patients that were sent for routine diagnostics was collected and subjected to the mPCR test. Subsequently, Decision tree (DT), random forest (RF), and support vector machine (SVM) hybrid diagnostic models were constructed and validated in a cohort of 99 women that included 74 BV patients and 25 healthy controls, and a separate cohort of 189 women comprising 75 BV patients, 30 intermediate vaginal microbiota subjects and 84 healthy controls, respectively.

**Results:**

The rate or abundance of *Lactobacillus crispatus* and *Lactobacillus jensenii* were significantly reduced in BV-affected patients when compared with healthy women, while *Lactobacillus iners*, *Gardnerella vaginalis*, *Atopobium vaginae*, BVAB2, *Megasphaera type* 2, *Prevotella bivia*, and *Mycoplasma hominis* were significantly increased. Then the hybrid diagnostic models were constructed and validated by an independent cohort. The model constructed with support vector machine algorithm achieved excellent prediction performance (Area under curve: 0.969, sensitivity: 90.4%, specificity: 96.1%). Moreover, for subjects with a Nugent score of 4 to 6, the SVM-BV model might be more robust and sensitive than the Nugent scoring method.

**Conclusion:**

The application of this mPCR test can be effectively used in key vaginal microbiota evaluation in women with BV, intermediate vaginal microbiota, and healthy women. In addition, this test may be used as an alternative to the clinical examination and Nugent scoring method in diagnosing BV.

## 1 Introduction

BV is one of the most common infectious diseases occurring in the female lower genital tract (FLGT) that can lead to adverse maternal outcomes (Javed et al., 2019; Ding et al., 2021). Timely and accurate clinical diagnosis is essential to improve patient prognosis and reduce antibiotic use. However, many limitations exist in the diagnostic methods currently used in clinical practice (Coleman and Gaydos, 2018). Multiplex quantitative PCR (mPCR) and next-generation sequencing (Fredricks et al., 2005; Coleman and Gaydos, 2018) have revolutionized our understanding of microbial communities by enabling complete and accurate vaginal microbiota profiling to determine the primary causative agent. These methods do not require microbial culture but involve direct extraction of genetic material from samples and the use of highly variable nucleic acid sequences to classify species, thereby constituting effective strategies for the comprehensive characterization of microbial diversity. Based on gene sequencing analysis, vaginal microbial communities were divided into separate categories determined by their composition, so-called community state type (CST I: *Lactobacillus crispatus*-dominated [*L. crispatus*], CST II: *Lactobacillus gasseri*-dominated [*L. gasseri*], CST III: *Lactobacillus iners*-dominated [*L. iners*], CST V: *Lactobacillus jensenii*-dominated [*L. jensenii*], CST IV: Anaerobic bacteria-dominated). Microbial communities dominated by *L. gasseri* (CST II) and *L. jensenii* (CST V) are less common than CST I and CST III and are considered opportune vaginal microbiota (Ravel et al., 2011; Abou Chacra et al., 2022). Moreover, the pathogenicity of different microorganisms in the host may be influenced by lifestyle and environmental factors, genetic susceptibility and other factors. However, due to the high costs and limited availability of NGS, the multiplex real-time PCR draws more attention (Kusters et al., 2015; Drew et al., 2020; Darie et al., 2022), but there is still a lack of reports on simultaneous quantitative detection of these four *Lactobacillus* spp. and BV-related pathogenic microorganisms (BVPs) by the mPCR method. Therefore, there is an urgent need to develop an mPCR assay that can comprehensively evaluate the key lactobacilli and BVPs in the FLGT, which will have important implications for the clinical management of BV in Chinese women of childbearing age.

**Figure 1:**
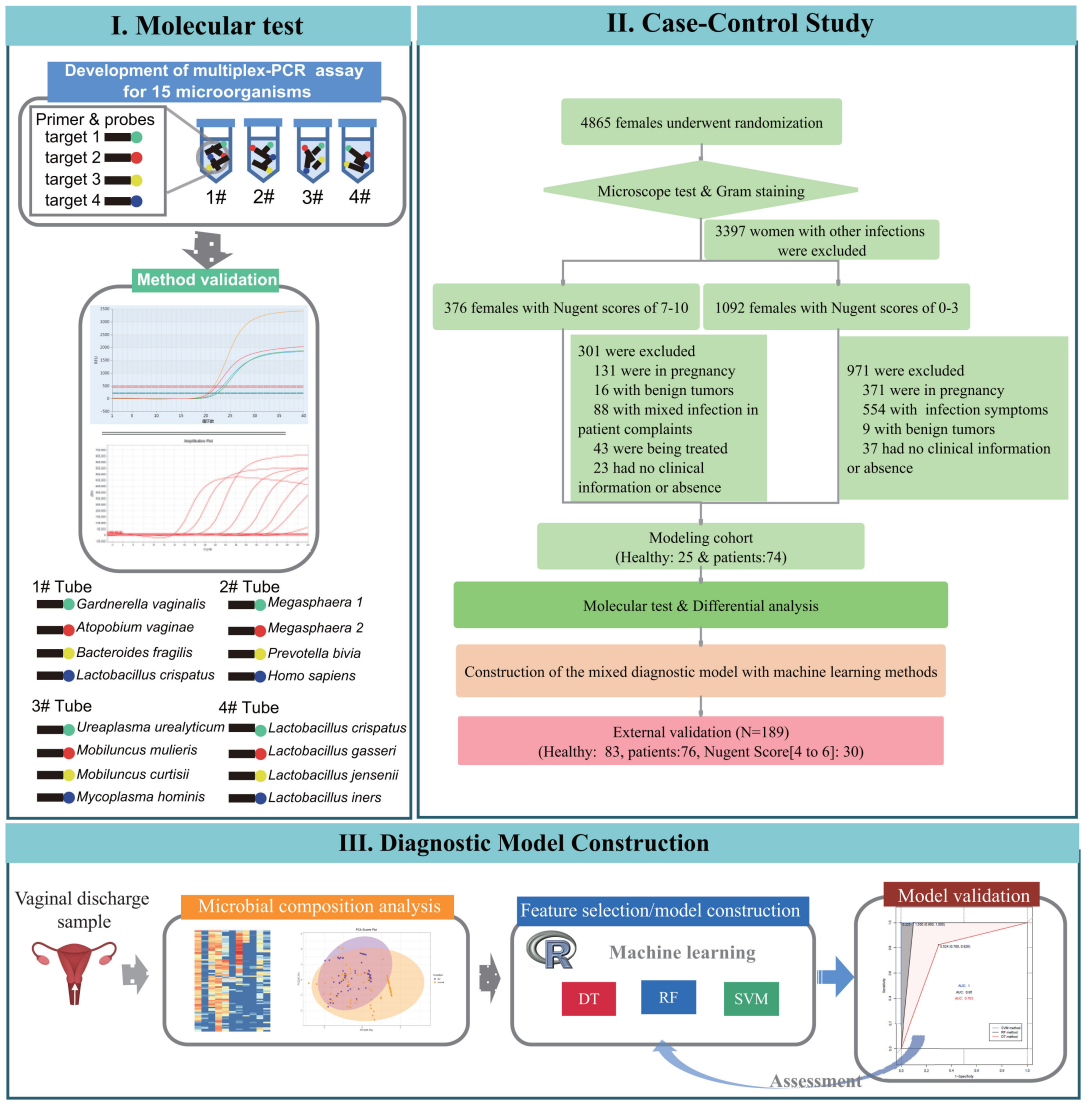
Flowchart of the three phases in this study: I. design and development process of species-specific 15-plex PCR assay, II. case-control studies for association study of bacterial vaginosis and candidate microbial markers selection for diagnostic of bacterial vaginosis, III. and development of machine learning diagnostic models and validation tests on clinical samples.

Thus, this study aimed to evaluate the microbial composition and dominant lactobacilli species in non-pregnant women with bacterial vaginosis using a multiplex PCR test and to determine diagnostic significance of its combination with artificial intelligence algorithms through a case-control study. A flow chart of our experimental design is shown in **Figure 1**.

## 2 Materials and methods

### 2.1 Study approval and sample collection

This study was approved by the Ethics Committee of Northwestern University (Xi’an, China) and the Ethics Committee of the First Affiliated Hospital of Xi’an Medical College (Xi’an, China) and was conducted in compliance with the ethical guidelines of the Declaration of Helsinki of the World Medical Association. We randomly collected vaginal secretion samples from 4865 subjects from the First Affiliated Hospital of Xi’an Medical College from January 2022 to April 2022. The inclusion criteria were at least 18 years old, not pregnant, no current use of hormonal or barrier contraceptive products, vaginal douching, tobacco or alcohol abuse, no hospitalization or systemic use of medication for chronic diseases or antibiotics/probiotics within the 6 months before sample collection, and no intercourse in the day before sampling. And then, smear microscopy, Gram staining and drying chemoenzymatic method (Bacterial vaginosis Test kit, BioPerfectus Co., Ltd. China) were used to determine the following parameters in accordance with the provided instructions: pH, flora density and diversity, Donders’ score, Nugent score (Nugent et al., 1991), Lactobacillus, Trichomonas, blastospore, catalase, cleanliness, leukocyte esterase, sialidase, and other relevant indicators. According to the test results, the samples were divided into the following types: (1) Normal flora: the density of vaginal flora was grade II-III, the diversity of vaginal flora was grade II-III, the dominant bacteria was Lactobacillus spp., no other pathogens were detected and the vaginal microbial function was normal. (2) Aerobic vaginitis (AV): Donders’ score ≥ 3 points, Hydrogen peroxide, leukocyte esterase and β-glucuronidase/coagulase positive can be used for auxiliary diagnosis. (3) Vulvovaginal candidiasis (VVC): Gram positive ovoid blastospores or tubular pseudohyphae were observed under the oil immersion objective. (4) Trichomonas vaginitis (TV): a large number of white blood cells with Trichomonas spp. were observed. (5) BV: Nugent score ≥ 7 points, Clue cells positive, PH ≥ 4.4 and Whiff test positive. (6) Intermediate Microbiota (IBV): the Nugent score was 4 to 6 points. After excluding 3397 cases with other reproductive tract infections (such as VVC, TV, etc.), the samples were divided into two groups according to Nugent score: the 7–10 group (376 cases) and the 0–3 group (1092 cases). In the 7–10 group, 131 pregnant women, 16 patients with malignant tumors, 88 patients with other non-reproductive tract infections, 43 patients who had received treatment, and 23 patients with missing information or without symptoms were excluded, and 74 symptomatic BV patients were retained as the BV group in the modeling cohort. The healthy group in the modeling cohort consisted of 25 non-pregnant, asymptomatic healthy women who underwent pre-pregnancy physical examination in the 0–3 group. Then, 25 healthy individuals (NO group) and 74 BV samples (BV group) in the modeling cohort were analyzed to screen candidate Lactobacilli and BVPs, and then the BV diagnostic model was constructed and internally validated. Finally, another vaginal secretion samples from 10607 subjects were randomly collected from the Affiliated Hospital of Xi’an Medical College from May 2022 to March 2023. And 83 healthy samples, 76 BV samples and 30 intermediate flora samples were obtained for external data validation according to the sample screening method described above. Clinical information and the data of the Laboratory for all participants were recorded in detail (**Supplementary Table S2**).

### 2.2 Vaginal sample collection and nucleic acid extraction

Two vaginal swabs per patient were sampled simultaneously. Sample secretions were collected from the lowest one third of the vagina by rotating the swabs (HCY Transsystem CY93050T swab, HCY technology, China) three times ensuring uniform distribution on both swabs. One sample was used for non-molecular tests, which performed at the laboratory of the First Affiliated Hospital of Xi’an Medical College. The second swab was used for the extraction and purification of microbial nucleic acids by The Upure Microbial DNA Kit^®^ (BioKeystone, Chengdu, China). Pure DNA was evaluated by the ratio of 260/280 with using NanoDrop 2000 (Thermo Scientific, Wilmington, DE, United States) and stored at −80°C for further use.

### 2.3 Primer and taqman probe design

Our qPCR methods have not been described elsewhere and were developed in-house. The whole genomic sequences of 11 BVPs (*Gardnerella vaginalis* [*G. vaginalis*], *Atopobium vaginae* [*A. vaginae*], *Bacteroides fragilis* [*B. fragilis*], BVAB2, *Prevotella bivia* [*P. bivia*], *Megasphaera type* 1 [*Megasphaera* 1], *Megasphaera type* 2 [*Megasphaera* 2], *Mobiluncus mulieris* [*M. mulieris*], *Mobiluncus curtisii* [*M.curtisii*], *Mycoplasma hominis* [*M. hominis*], and *Ureaplasma urealyticum* [*U. urealyticum*]) and 4 lactobacilli (*L. crispatus*, *L. gasseri*, *L. jensenii*, and *L. iners*) were downloaded from the NCBI database. MAFFT software was used for global sequence alignment to screen-conserved sequences. Primers were designed using the Primer-BLAST (https://www.ncbi.nlm.nih.gov/tools/primer-blast/) online tool according to the primer design principles and commissioned to be synthesized by Bioengineering (Shanghai, China) Co., Ltd. The PCR reaction employed universal primers for the host gene (*β*-actin) to provide the internal positive control. The specificity of primers and probes were verified by sanger sequencing of the PCR products.

### 2.4 Development of the BVLaB assay

We used four independent reactions to achieve simultaneous detection of 15 microbial targets, with four targets per reaction. Probes were labeled with different fluorescent molecules.The mPCR amplification was performed using ABI 7500 platform (Applied Biosystems, Wilmington, DE, United States) in 20 μL of reaction mixture solution containing 5 μL of nucleic acid template, 4 μL of 5×buffer (FAPON Biotech Inc., Guangzhou, China), 2 μL of 10×solution I (FAPON Biotech Inc., Guangzhou, China), 1 μL of dNTP mix (FAPON Biotech Inc., Guangzhou, China), and 0.275 μL of Hotstart Hitaq polymerase mix (FAPON Biotech Inc., Guangzhou, China), and sterilized distilled water. The reaction conditions were as follows: 95°C for 5 min, followed by 40 cycles of 95°C for 50 s, and 60°C for 40 s.

### 2.5 Construction and validation of diagnostic model

Univariate analysis was used to compare the characteristics of 15 microorganisms between the healthy and the BV groups, and to screen indicators that could be used to construct diagnostic models. The differential factors or high-resolution indicators (sensitivity or specificity higher than 80%) were used as candidate features for model construction. Three machine learning algorithms, support vector machine (SVM), decision tree (DT), and random forest (RF) algorithm, were used for the construction of mixed diagnostic models of BV. The modeling cohort were randomly divided into a training dataset and a validation dataset in a ratio of 2:1 in the modeling process. We then internally validated our whole model development strategy using the validation dataset of modeling cohort. In addition, an independent validation dataset of 159 samples was used to verify the performance of diagnostic models. The predictive performance of diagnostic models was evaluated by a series of evaluation metrics, including area under the curve (AUC), sensitivity and specificity.

### 2.6 Statistical analysis

Quantification cycle (Ct) values obtained by the mPCR were utilized for subsequent data analysis, with a uniform assignment of 40 to microorganisms that were either undetected or had Ct values exceeding the maximum quantification cycle. A lower Ct value indicated a higher abundance of this organism in the sample. Data preprocessing, model constuction and statistical analysis were performed using perl (www.perl.org) and R (https://www.r-project.org/) software. The Kolmogorov–Smirnov and Shapiro–Wilk tests were used to test the normality of the data distribution. ANOVA, Pearson’s chi-squared test, and Fisher’s exact test were used to compare continuous and categorical data. Feature selection and diagnostic performance evaluation of the models were performed by differential analysis and ROC analysis, respectively. Statistical analysis were only performed for data with a sample size of more than 20 cases. A *p* value less than 0.05 was considered statistically significant.

## 3 Results

### 3.1 Development and performance evaluation of the BVLaB assay

**Figure 2:**
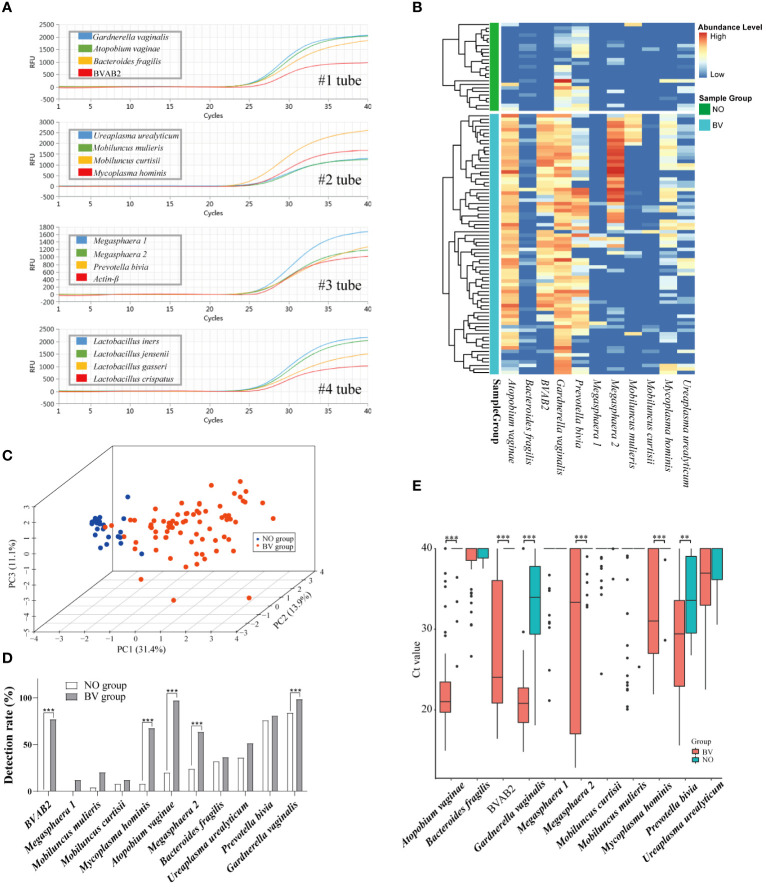
Amplification plots of 15 microorganisms using the new mPCR assay and the distribution pattern of 11 BV pathogenic microorganisms (BVPs) in vaginal discharges. **(A)** Amplification results of the 15 targets by the mPCR assay. **(B)** Heatmap displays differential abundance of 11 BVPs between healthy women and BV patients. **(C)** Relationships among samples visualized by principal component analysis. And **(D, E)** represents the differences of detection rate and abundance between healthy women and BV patients. Ct, Quantification cycle values in PCR. **, *p*<0.01, ***, *p*<0.001.

Species-specific primers designed manually from variable regions are summarized in **Supplementary Table S1**. In order to assess the sensitivity of the BVLaB assay, the detection limit of the mPCR was performed with different dilution of the template. As shown in **Figure 2A** and **Supplementary Table S1**, the newly developed method was able to amplify all targets in a range of 10^3^–10^8^ copies μL^−1^ with a strong linear relationship, and all 15 targets were detectable even as DNA template amounts was as low as 15 copies μL^−1^. In addition, non-specific amplification of other organisms or non-template agents was undetected by the BVLaB assay. These results indicate that the new assay was proved to be highly sensitive for amplification of low quantities of DNA and thus could be applied to detect pathogenic microorganisms.

### 3.2 Population characteristics and molecular testing results

A total of 288 samples were collected for differential analysis and model construction in this study. Information regarding the clinical samples is summarized in the **Table 1**. There were significant differences in the detection rates of 15 microorganisms in the samples. Overall, the detection rate of each microorganism ranged from 9.09% to 94.95%. Among BVPs, the detection rate of *G. vaginalis* was the highest (94.95%), followed by *A. vaginae* (77.78%), and that of *Megasphaera* 1 was the lowest (9.09%). Among the four species of *Lactobacillus* spp., *L. iners* could be detected in the largest number of samples, with the detection rate of 85.86% and 75.29% in the Lactobacilli cohort and modeling cohort, respectively, followed by *L. crispatus* (64.7% and 41.41% in the Lactobacilli and modeling cohort, respectively).

**Table 1:** Clinical and laboratory statistics collected from the modeling and validation datasets.

Characteristics	Modeling cohort	Validation cohort
Number of samples (n)^a^		
Healthy group	25	83
BV group	74	76
intermediate microbiota group (NS 4~6)	/	30
Number of samples for diagnostic model construction (n)		
Training datasets	15 (Healthy) / 51 (BV)	/
Validation datasets	10 (Healthy) / 23 (BV)	83 (Healthy) / 76 (BV)
Age at diagnosis (n. %)^b^		
15~24	14 (14.14)	16 (8.47)
25~34	48 (48.48)	98 (51.85)
35~44	25 (25.25)	56 (29.63)
45~70	12 (12.12)	20 (10.58)
Detection rate of microbes (n. %)		
*Lactobacillus crispatus*	41 (41.41)	104 (55.03)
*Lactobacillus gasseri*	24 (24.24)	89 (47.09)
*Lactobacillus jensenii*	15 (15.15)	82 (43.39)
*Lactobacillus iners*	85 (85.86)	164 (86.77)
*Atopobium vaginae*	77 (77.78)	140 (74.07)
*Bacteroides fragilis*	35 (35.35)	35 (18.52)
*BVAB2*	57 (57.58)	88 (46.56)
*Gardnerella vaginalis*	94 (94.95)	165 (87.3)
*Prevotella bivia*	79 (79.8)	162 (85.71)
*Megasphaera 1*	9 (9.09)	105 (55.56)
*Megasphaera 2*	53 (53.54)	96 (50.79)
*Mobiluncus mulieris*	16 (16.16)	48 (25.4)
*Mobiluncus curtisii*	11 (11.11)	64 (33.86)
*Mycoplasma hominis*	52 (52.53)	143 (75.66)
*Ureaplasma urealyticum*	47 (47.47)	116 (61.38)

^a^n, ^b^% represent the number of samples and percent of total samples, respectively.

NS, Nugent score; BV, bacterial vaginosis. NO group, healthy group of modeling cohort or validation cohort.

### 3.3 Alteration of BVPs distribution in vaginal discharges

To determine whether the distribution of 11 BVPs were associated with healthy women and BV patients, 99 samples (NO = 25 and BV = 74) were independently tested by the assay in the modeling cohort. As shown in **Figures 2B–E**, the heatmap plot displayed the alteration of BVPs’ distribution between the two groups. And the PCA results indicated a significant difference in the distribution of the first two principal components between the two groups of samples. *P. bivia* (76.0%) and *G. vaginalis* (84.0%) were detected in more than half of the samples in the healthy group, while seven microbes (*G. vaginalis* [98.6%], *P. bivia* [81.1%], *A. vaginae* [97.3%], BVAB2 [77.0%], *M. hominis* [67.6%], *Megasphaera* 2 [63.5%], and *U. urealyticum* [51.4%]) were detected in the BV group. Five BVPs were detected more frequently in the BV group than in the healthy group: *A. vaginae* (*p* < 0.0001), BVAB2 (*p* < 0.0001), *Megasphaera* 2 (*p* = 0.001), *M. hominis* (*p* < 0.0001), *G. vaginalis* (*p* = 0.0138). As expected, these six BVPs were present in higher abundance in the BV group than in the healthy group: *A. vaginae* (*p* ≤ 0.0001), BVAB2 (*p* ≤ 0.0001), *Megasphaera* 2 (*p* ≤ 0.0001), *M. hominis* (*p* ≤ 0.0001), *G. vaginalis* (*p* = 0.0138), *P. bivia* (*p* = 0.0039).

### 3.4 Alteration of Lactobacilli distribution in vaginal discharges

**Figure 3:**
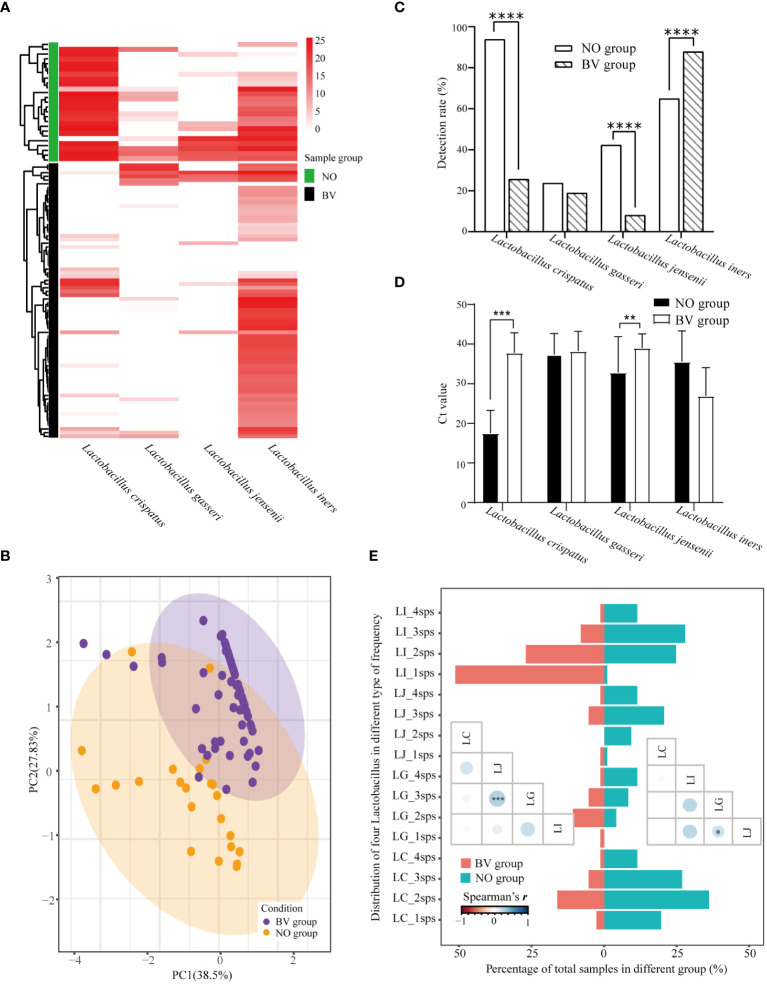
Distribution pattern of four species of *Lactobacillus* spp. in vaginal discharges. **(A)** Heatmap of hierarchical clustering analysis showed differential abundance of four Lactobacilli between healthy women and BV patients. **(B)** Relationships among samples visualized by principal component anlaysis. And **(C, D)** represented the differences of detection rate and abundance between healthy women and BV patients. **(E)** Barplot and the lower triangular correlation matrix displays the co-detection of four species of *Lactobacillus* spp. LC_1sps represented only *Lactobacillus crispatus* was detected in the sample, and LC_2sps represented *Lactobacillus crispatus* and one of the other Lactobacilli were detected in the sample, etc. LI, *Lactobacillus iners*; LJ, *Lactobacillus jensenii*; LG, *Lactobacillus gasseri*; LC, *Lactobacillus crispatus*. Ct, Quantification cycle values in PCR. *, *p*<0.05. **, *p*<0.01, ***, *p*<0.001. ****, *p*<0.0001.

*Lactobacillus* spp. is closely associated with the maintenance of a stable microenvironment in the FLGT. As presented in **Figure 3A**, There were statistically significant differences in the distribution of *L. crispatus* and *L. jensenii* in the NO and BV groups. The PCA results demonstrated that the distribution features of these four species of *Lactobacillus* spp. can be used to distinguish whether the subjects are BV positive (**Figure 3B**). The detection rate of *L. iners* was higher in the BV group than in the NO group, whereas that of *L. crispatus* and *L. jensenii* showed an opposite trend (**Figure 3C**). The abundance of *L. crispatus* and *L. jensenii* in the BV group was significantly lower than that in the NO group (Both *p* ≤ 0.0001) (**Figure 3D**). In addition, coexistence analysis (**Figure 3E**) showed that *L. iners* was the most predominant type in the BV group, mainly in samples with one and two species of Lactobacillus detected. There is a significant correlation between *L. jensenii* and *L. gasseri*, which may be related to the low detection rate of these two lactobacilli (**Figure 3E**).

### 3.5 Correlation analysis between lactobacilli and BVPs

**Figure 4:**
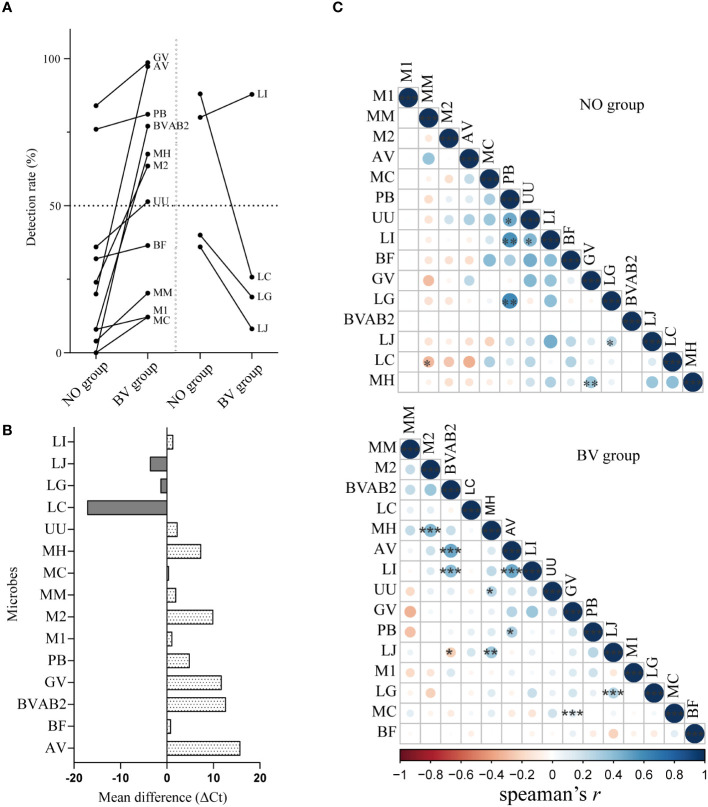
Correlation analysis between key lactobacilli and BV-related microorganisms in vaginal microenvironment. **(A, B)** represented the changes of detection rate and abundance of 15 microorganisms between healthy women and BV patients. **(C)** The lower triangular correlation matrix showed the correlations between the distribution of 15 microorganisms in the BV and healthy group. Mean difference represents the difference of Ct value of a microorganism between BV group and healthy group, and the positive value represents the content of the index in the healthy group is lower than that in the BV group. GV, *Gardnerella vaginalis*; AV, *Atopobium vaginae*; BF, *Bacteroides fragilis*; BVAB2, Bacterial vaginosis–associated bacteria 2; UU, *Ureaplasma urealyticum*; MM, *Mobiluncus mulieris*; MC, *Mobiluncus curtisii*; MH, *Mycoplasma hominis*; M1, *Megasphaera* 1; M2, *Megasphaera* 2; PB, *Prevotella bivia*; LI, *Lactobacillus iners*; LJ, *Lactobacillus jensenii*; LG, *Lactobacillus gasseri*; LC, *Lactobacillus crispatus*. *, *p*<0.05. **, *p*<0.01, ***, *p*<0.001.

The similarity in the distribution of microorganisms in different infection states might be used to predict the interactions between microorganisms. The detection rates and abundance levels of *L. iners* and all BVPs were higher in the BV group than in the NO group (**Figures 4A, B**). As expected from **Figure 4C**, we found positive correlations between the abundance of *L. iners* and some BVPs in both groups, but the microbial species of BVPs associated with *L. iners* were not consistent between the two groups. For example, in the NO group, the abundance of *P. bivia* and *U. urealyticum* showed significant positive correlations with *L. iners*, while *L. iners* was found to have a significant positive correlation with BVAB2 and *A. vaginae* in the BV group, which may indicate that *L. iners* may be an independent factor in the development of BV. In addition, we also found that there were correlations between various BVPs in the BV group, such as a significant positive correlation ermerged between the abundance of *A. vaginae* and *P. bivia*, *G. vaginalis* was also significantly positively correlated with *M.curtisii*, suggesting the synergistic effect of these BVPs on the occurrence of BV.

### 3.6 Candidate indicators for BV diagnosis

Given the observed differences in the distribution of microorganisms between healthy individuals and BV patients, the performance of the developed method for detecting and measuring abundance as a diagnostic indicator for each microorganism was evaluated (**Table 2**). When the presence (Ct < 40) or absence (Ct ≥ 40) of microorganisms was used as the BV diagnostic criteria, A. *vaginae* showed the best diagnostic performance (sensitivity 91%, specificity 94%), Considering the influence of age on the microbial community of the vaginal microenvironment, we further divided the samples into different age subgroups for analysis, and the results showed that BVAB2 and *A. vaginae* had the best diagnostic performance in the age group of 25–34 (sensitivity 91%, specificity 100%) and 35–45 (sensitivity 75%, specificity 95%), respectively. Moreover, the ROC analysis was then performed based on the abundance of each microorganism (Ct value) and the cut-off value (CoV) with the maximum *Youden’ s index* was used as the BV diagnostic criteria, *L. crispatus* showed the best diagnosis performance (CoV: 21.24, sensitivity 97%, specificity 94%) in the modeling cohort and the age group of 25–34 (CoV: 21.55, sensitivity 100%, specificity 94%), while *A. vaginae* performed best in the age group of 35–44 (CoV: 29.68, sensitivity 95%, specificity 100%). These results indicated that the presence and abundance of microorganisms can be used as candidate markers for the diagnosing BV, but the influence of age should still be considered in the diagnosis.

**Table 2:** The results of performance evaluation of each microorganism of detection status as a diagnostic indicator for BV.

Index^a^	Modeling cohort (Total samples)				Modeling cohort (Samples of 25–34 age group)				Modeling cohort (Samples of 35–44 age group)			
	Cut-off value^#^	Sensitivity(%)	Specificity(%)	*Youden’s index^##^*	Cut-off value	Sensitivity(%)	Specificity(%)	*Youden’s index*	Cut-off value	Sensitivity(%)	Specificity(%)	*Youden’s index*
LC	≤40	8	17	-0.75	≤40	13	12	-0.75	≤40	12	20	-0.68
LG	≤40	55	38	-0.07	≤40	68	27	-0.05	≤40	54	38	-0.08
LJ	≤40	45	13	-0.42	≤40	58	0	-0.42	≤40	43	24	-0.33
LI	≤40	79	51	0.3	≤40	85	37	0.22	≤40	82	53	0.35
AV	≤40	91	94	0.85	≤40	100	88	0.88	≤40	75	95	0.7
BF	≤40	27	77	0.04	≤40	41	56	-0.03	≤40	17	86	0.03
BVAB2	≤40	60	100	0.6	≤40	91	100	0.91	≤40	50	100	0.5
GV	≤40	80	78	0.58	≤40	100	61	0.61	≤40	67	91	0.58
PB	≤40	30	76	0.06	≤40	56	62	0.18	≤40	25	86	0.11
M1	≤40	28	100	0.28	≤40	49	100	0.49	≤40	17	100	0.17
M2	≤40	41	89	0.3	≤40	55	79	0.34	≤40	33	89	0.22
MM	≤40	29	94	0.23	≤40	44	100	0.44	≤40	18	88	0.06
MC	≤40	26	82	0.08	≤40	42	60	0.02	≤40	18	100	0.18
MH	≤40	49	96	0.45	≤40	59	94	0.53	≤40	44	100	0.44
UU	≤40	31	81	0.12	≤40	24	52	-0.24	≤40	19	89	0.08
LC	≥21.24	97	94	0.91	≥21.55	100	94	0.94	≥20.64	95	92	0.87
LG	≥33.18	91	20	0.11	≥32.99	93	19	0.12	≥34.36	95	19	0.14
LJ	≥35.94	93	42	0.35	≥38.61	100	38	0.38	≥23.78	100	42	0.42
LI	≤37.75	88	35	0.23	≤36.1	86	38	0.24	≤38.27	90	35	0.25
AV	≤30.29	91	96	0.87	≤28.66	93	95	0.88	≤29.68	95	100	0.95
BF	≤0	1	0	0	≤40	68	35	0.03	≤38.74	81	25	0.06
BVAB2	≤38.22	77	100	0.77	≤38.22	93	100	0.93	≤36.91	81	100	0.81
GV	≤26.73	93	92	0.85	≤26.64	100	90	0.9	≤28.4	90	100	0.9
PB	≤27.63	42	96	0.38	≤26.58	50	100	0.5	≤33.21	81	100	0.81
M1	≤0	100	0	0	≤0	100	0	0	≤0	100	0	0
M2	≤32.5	49	100	0.49	≤36.53	54	85	0.39	≤35.77	76	100	0.76
MM	≤0	1	0	0	≤0	100	0	0	≤28.63	76	25	0.01
MC	≤0	1	0	0	≤36.87	96	5	0.01	≤0	100	0	0
MH	≤37.9	68	96	0.64	≤38.48	54	95	0.49	≤38.6	76	100	0.76
UU	≤37.66	51	72	0.23	≤37.64	61	65	0.26	≤0	100	0	0

a GV, Gardnerella vaginalis; AV, Atopobium vaginae; BF, Bacteroides fragilis; BVAB2, BVAB2; UU, Ureaplasma urealyticum; MM, Mobiluncus mulieris; MC, Mobiluncus curtisii; MH, Mycoplasma hominis; M1, Megasphaera 1; M2, Megasphaera 2; PB, Prevotella bivia; LI, Lactobacillus iners; LJ, Lactobacillus jensenii; LG, Lactobacillus gasseri; LC, Lactobacillus crispatus.

#Cut-off value represent the dividing points where the subjects are divided into different categories: BV, or Non-BV.

##The Youden’ s index (or Youden’ s J statistic) is defined as J = sensitivity + specificity − 1.

### 3.7 Model construction and performance evaluation

**Figure 5:**
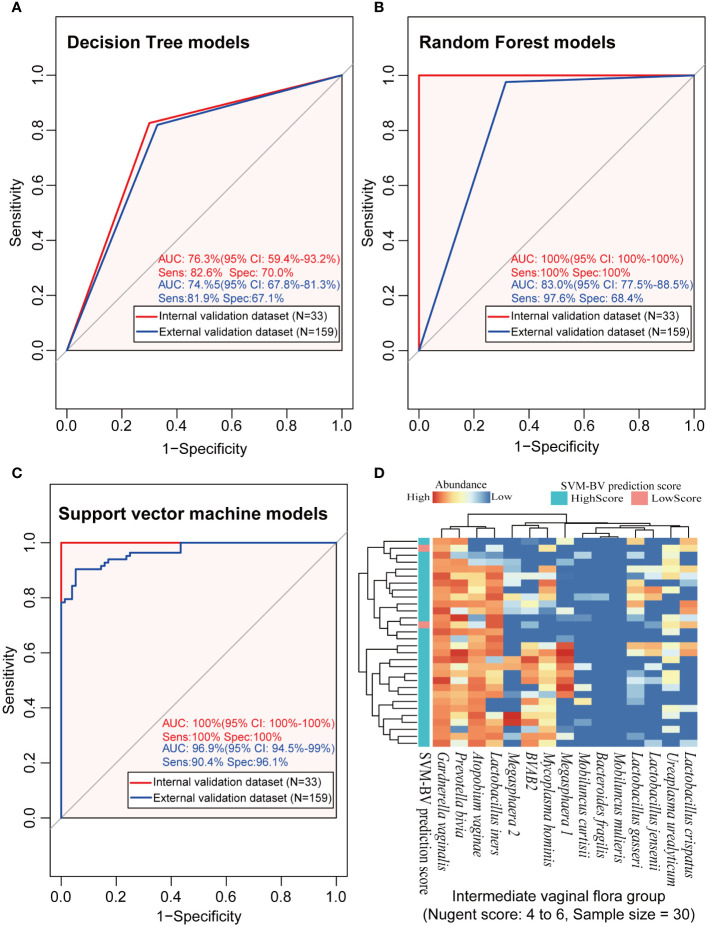
The analysis results of the three machine learning models in the modeling, external validation and intermediate microbiota dataset. **(A–C)** represented the performance of the BV diagnostic models constructed by the three machine learning algorithms in the internal and external validation datasets, and **(D)** represented the prediction results using the constructed SVM model on the intermediate vaginal flora samples. ROC, receiver operating characteristic; AUC, the area under the curve; Sens, sensitivity; Spec, specificity; CI, 95% confidence interval; DT, decision tree algorithm; RF, random forest algorithm; SVM, support vector machine algorithm.

Given our findings on microbial distribution patterns in BV patients, three lactobacilli (*L. crispatus*, *L. jensenii*, and *L. iners*), seven BVPs (G. vaginalis, *A. vaginae, Megasphaera* 2, *P. bivia, M. mulieris and M. hominis*) and age of the subjects were used for modeling. As shown in **Figure 5**, the diagnostic AUC of the DT model was 0.736 (95% CI: 0.594–0.932) in the modeling cohort, with the sensitivity and specificity of 82.6% and 70.0%, respectively. The diagnostic AUC, sensitivity, and specificity of the SVM and RF models were all 100%. In the external validation dataset, the diagnostic AUC of the DT was 0.745 (95% CI: 0.678–0.813), with the sensitivity and specificity of 81.9% and 67.1%, respectively. The diagnostic AUC of the RF model was 0.830 (95% CI: 0.775–0.885), with the sensitivity and specificity of 97.6% and 68.4%, respectively. The SVM model had a diagnostic AUC of 0.969 (95% CI: 0.945–0.99), with the sensitivity and specificity of 90.4% and 96.1%, respectively. Overall, the SVM algorithm resulted in the highest performance in terms of AUC value in the internal validation and external validation datasets among the other two models, proving that the SVM model has more potential to diagnose BV.

Further investigation of the diagnostic consistency with intermediate scores using the SVM model is presented in **Figure 5D**. The heatmap results showed that 93.3% (28/30) of the IBV samples were also identified as BV positive by the new method, but the diagnosis of the other 6.7% (2/30) of the intermediate BV samples were different between the two methods.

## 4 Discussion

Several previous studies have reported the high sensitivity and accuracy of mPCR methods for the diagnosis of BV (Cartwright et al., 2012; Hilbert et al., 2016; Gaydos et al., 2017; Coleman and Gaydos, 2018; van der Veer et al., 2018; Carter et al., 2023). For example, The Allplex bacterial vaginosis assay, a multiplex PCR-based test for BV based on quantitative results, has been reported for Lactobacillus spp., *G. vaginalis*, and *A. vaginae* with qualitative detection of *Megasphaera 1*, *B. fragilis*, BVAB2, and *Mobiluncus* spp., with a sensitivity and specificity of 65% and 98%, respectively (Drew et al., 2020). Kusters et al. also developed a semiquantitative multiplex PCR assay for five microorganisms (*G. vaginalis*, *A. vaginae*, *Megasphaera* 1, *L. crispatus*, and *L. iners*) for BV diagnosis (Kusters et al., 2015). The FDA-approved nucleic acid based diagnostic tests for BV, such as BD MAX™ Vaginal Panel (Becton,Dickinson and Company, United States) and Aptima^®^ BV Assay(Hologic, Inc., San Diego, CA), also consist of *Lactobacillus spp* (2 to 3 kind of species) and several BVPs, both of which have high sensitivity but moderate specificity. These studies suggested that Lactobacilli are a good indicator of the changes in the vaginal microenvironment as pathogenic bacteria. Moreover, in addition to the known BV related pathogens such as *G. vaginalis* and *A. vaginae*, a high proportion of genital mycoplasma (*M. hominis* and *U. urealyticum*) infections have been found in patients with BV, and some studies have shown that these genital mycoplasma infections are also related to the drug resistance of BV (Lendamba et al., 2022; Rosário et al., 2023). However, none of the previous methods could reflect the changes in the levels of the representative species of five CSTs at the same time. Therefore, we developed a general-purpose fluorescence PCR instrument-based BVLaB assay for key microorganisms in the five CSTs or microorganisms with high detection rate reported in previous studies (Kinoshita et al., 2014; Javed et al., 2019; Lendamba et al., 2022; Li et al., 2022; Carter et al., 2023).

Similar to previous reports (Gryaznova et al., 2022; Rak et al., 2022; Roachford et al., 2022), the detection rates and/or abundance of *G. vaginalis*, *P. bivia*, *A. vaginae*, BVAB2, *Megasphaera* 2 (not *Megasphaera* 1), and *M. hominis* changed significantly during the transition from the healthy state to the diseased state. These results may indicate a high abundance of *G. vaginalis* and *P. bivia* in the FLGT and suggest that these two bacteria act as early colonizers, whereas *A. vaginae* and other BV-associated bacteria are secondary colonizers, and that these early colonizers may evade the immune system while forming a bacterial vaginosis biofilm (Muzny et al., 2019; Randis and Ratner, 2019; Muzny et al., 2020).

Significant differences in the genomic characteristics and metabolic processes among the four lactobacilli may lead to their different roles in the vaginal microenvironment. A genome-wide study reported that *L. iners* has a smaller genome sequence than *L. crispatus*, *L. jensenii*, and *L. gasseri* (Kwak et al., 2020; Zheng et al., 2021). *L. crispatus*, *L. jensenii*, and *L. gasseri* are the main D (–) isomer lactic acid-producing microorganisms (Edwards et al., 2019; Kerry-Barnard et al., 2022), whereas *L. iners* mainly produces L (–) isomer lactic acid (van Houdt et al., 2018; Witkin et al., 2019), which might lead to the differences in host protection and inhibition of pathogen colonization in the CSTs dominated by these microorganisms. In this study, the difference in the distribution of *L. crispatus* and *L. iners* between healthy status and diseased status was similar to that reported in previous studies (Ravel et al., 2011; Javed et al., 2019). In addition, we found a significant positive correlation between the abundance of LI and the abundance of multiple BVPs, indicating that the increase in *L. iners* in the vaginal microenvironment is related to the increased risk of BV (Nilsen et al., 2020; Zhu et al., 2022). The differential distribution of these microorganisms suggests that they have a potential to serve as molecular markers for BV diagnosis.

The “grey zones” of the intermediate Nugent score has been one of the most controversial issues in the diagnosis of BV. Sometimes, in these cases, some of the symptomatic relapses were due to worsening of the preexisting underdiagnosed dysbiosis state rather than BV relapse. For example, Campisciano et al. showed that only 17 (16.7%) of 102 women diagnosed as intermediate BV by Gram’s stain were confirmed by qPCR as an intermediate clinical picture (partial BV) (Campisciano et al., 2021). In our study, we also observed a misdiagnosis of the intermediate microbiota by the Gram staining, which was not confirmed by the SVM-based qPCR method. These results may indicate that molecular techniques can better elucidate qualitative and quantitative changes in the vaginal microbiota, helping clinicians decipher some of the “grey zones” in clinical practice.

Furthermore, as age impacted prediction accuracy, we constructed BV diagnostic models based on differences in microbial distribution patterns and found that the SVM algorithm outperformed the other two modeling strategies. The SVM algorithm model is more robust than models built using other algorithms, and this advantage is confirmed by the performance evaluation metrics in the results of cross-validation. Although some studies have shown that machine learning algorithms have great potential in building and optimizing risk assessment and diagnosis models, there are still some barriers to the application of such techniques in clinical settings, such as the lack of easy interpretation and instability (Hardy et al., 2021; Song and Lee, 2022).

The present study still has some limitations. Although the distribution characteristics of four lactobacilli and several key BVPs in the vaginal secretions of healthy women and BV patients have been identified, the sample size used in this study is still relatively small; And the patient population was exclusively Chinese women and the results may not be generalizable to other racial groups as the microbiome may differ in both normal and disease states among different groups. Therefore, this is an exploratory and preliminary study, and further studies with larger sample sizes are needed to confirm these results. Moreover, the stability and accuracy of the model developed using machine learning algorithms are closely related to the improvement in the sample size, and more data support can help eliminate the interference of potential confounding and nonrandom factors in the model. However, the synergistic, antagonistic, and additive effects of vaginal microorganisms in the transition from a healthy state to the diseased state are still not clear. Therefore, we propose to overcome this limitation in further studies with larger sample sizes. Furthermore, our subsequent work will focus on the underlying causes of microbial changes and abnormalities during different stages of disease progression.

## 5 Conclusion

In conclusion, a highly sensitive, specific, indigenous, single-run mPCR for BV diagnosis has been developed, which can simultaneously detect *L. crispatus*, *L. gasseri*, *L. jensenii*, *L. iners* and 11 key BVPs. And we preliminary study provided information about the distribution characteristics of BV-associated microorganisms in the vaginal secretions of healthy women and BV patients, combined with machine learning algorithms to construct a diagnostic model of BV, which may contribute to understanding the dynamic change of the microbial community of BV and provided a rapid, comprehensive, and accurate diagnostic strategy.

## Data availability statement

The original contributions presented in the study are included in the article/**Supplementary Material**. Further inquiries can be directed to the corresponding authors.

## Ethics statement

The studies involving humans were approved by the Ethics Committee of Northwestern University. The studies were conducted in accordance with the local legislation and institutional requirements. The participants provided their written informed consent to participate in this study.

## Author contributions

SL: Conceptualization, Methodology, Writing – original draft, Writing – review & editing. ZL: Conceptualization, Methodology, Writing – original draft, Writing – review & editing. XC: Conceptualization, Methodology, Writing – original draft. FC: Writing – review & editing. HY: Conceptualization, Methodology, Writing – original draft. XS: Data curation, Formal Analysis, Writing – review & editing. YC: Data curation, Formal Analysis, Writing – original draft. LW: Conceptualization, Methodology, Project administration, Resources, Writing – review & editing. PD: Investigation, Methodology, Project administration, Resources, Writing – review & editing.
