# Supplementary material for: Vaginal microbiota molecular profiling and diagnostic performance of artificial intelligence-assisted multiplex PCR testing in women with bacterial vaginosis: a single-center experience
Source: Front Cell Infect Microbiol. 2024 Apr 5;14:1377225. doi: 10.3389/fcimb.2024.1377225 (PMC11026559; doi:10.3389/fcimb.2024.1377225)
Supplement: Supplementary file 1 [file Table_1.docx]

**Supplemental Table 1**. Information sheet of target sequence, primer design, and assay performance of the BVLaB assay.

| **Number of reaction tube** | **Targets** | **Primer sequences**  **(5'-3')** | **Reference sequence**  **(NCBI ID)** | **Usage of PCR**  **(μL·10μM)** | **Limit of detection**  **(copies·μL^-1^)** | **Linear detection range (copies·μL^-1^)** | ***R*^2^** |
| --- | --- | --- | --- | --- | --- | --- | --- |
| 1 | *Gardnerella vaginalis* | F：CAAAGCACCGTGACGTTCAAT  R：CCCCACTACAGATGCAAC  P：FAM-CTCCAAATGCAATGAAGCTGC-BHQ1 | LR134385.1 | F & R^a^: 0.5  P^a^: 0.25 | 11.75 | 4.70×10^2^ to 4.70×10^9^ | 0.988 |
|  | *Atopobium vaginae* | F：CGTCCATATGAAGAAGCTTAAAC  R：CATGTTCGTAGGGGATTGTGTA  P：ROX-CTGGACATGGATACGCAGCGT-BHQ2 | CP065631.1 | F & R: 0.5  P: 0.25 | 11.67 | 4.67×10^2^ to 4.67×10^8^ | 0.990 |
|  | *Bacteroides fragilis* | F：TTCCAGTGAGGTCCTCTCAGGTAC  R：GAGCTAACACAATCGTACTGATGCT  P：CY5-CATAAAATCTGACAATCGACGCAGTCT-BHQ2 | CP036555.1 | F & R: 0.5  P: 0.5 | 11.70 | 4.68×10^2^ to 4.68×10^8^ | 0.982 |
|  | *BVAB2* | F：ACAGTTTCTAATGATCCCAAGT  R：GGGCGTACCGGGCATCTGTT  P：VIC-CAGGTAGATACAGTGTGATGT-MGB | MF671208.1 | F & R: 0.5  P: 0.25 | 11.67 | 4.67×10^2^ to 4.67×10^8^ | 0.991 |
| 2 | *Ureaplasma urealyticum* | F：CAGCTCGGTTAATTGCTCCTGG  R：AAGGTAATGATTCGGTGTCAGGATCA  P：FAM-CTAATTTAGTTGGAGTGAATTCGT-MGB | CP039963.1 | F & R: 0.5  P: 0.5 | 11.67 | 4.67×10^2^ to 4.67×10^8^ | 0.981 |
|  | *Mobiluncus mulieris* | F: GCTGACGAAGCGAAATCCA  R: CAGAGCCGAAGTTTTACCC  P: ROX-CAGGAGCCGAAGTTTTAAGG-MGB | NZ_VTAH00000000.1 | F & R:0.5  P: 0.25 | 11.67 | 4.67×10^2^ to 4.67×10^8^ | 0.986 |
|  | *Mobiluncus curtisii* | F：CACGCGGACCTTCTCCG  R：TGGCAGCGATTAAGGAACTGAT  P：CY5-CAAGTCAGTGTTACATGGCT-MGB | CP068112.1 | F & R: 0.5  P: 0.5 | 11.82 | 4.73×10^2^ to 4.73×10^8^ | 0.979 |
|  | *Mycoplasma hominis* | F：TTCACTAAACGGGTATTTTTACACGT  R：TGGCTATATTGCATGAAGTGCTT  P：VIC-ATCCGACAGTATTAGGATATTGGT-MGB | CP055151.1 | F & R:1  P: 0.5 | 11.52 | 4.61×10^2^ to 4.61×10^8^ | 0.983 |
| 3 | *Megasphaera 1* | F：CGCCCTTACGACGCCAATA  R：ATGCCAACGTATCCAATCCG  P：FAM-CGGACAACGTTGCCACGTTACGATTA-MGB | NR_181404.1 | F & R: 1  P: 0.5 | 11.47 | 4.59×10^2^ to 4.59×10^8^ | 0.990 |
|  | *Megasphaera 2* | F：AGTTAGCACGTGCTTAATCTCTAC  R：CTCTGTTATCGAGGATCGAAGG  P：ROX-TACCGCACTCATATGTGGCTATTACACC-BHQ2 | NR_178779.1 | F & R: 0.5  P: 0.25 | 11.72 | 4.69×10^2^ to 4.69×10^8^ | 0.994 |
|  | *Prevotella bivia* | F：CAAGATGCTCCGCGATTATGTATA  R：CGGATCAATACTCTAGCCGATAACT  P：CY5-ACTATGCCACCGATTGCTTCACCTC-BHQ2 | NZ_JH660658.1 | F & R: 0.5  P: 0.25 | 11.67 | 4.67×10^2^ to 4.67×10^8^ | 0.986 |
|  | *Homo sapiens* | F：CACTCCAAGCCGAGCTTTAA  R：GACAGATTGTGGTCGTAGGT  P：VIC-TGGCACTTGTACATGACCA-BHQ2 | AY582799.1 | F & R: 0.5  P: 0.5 | 2.50 | 1×10^2^ to 1×10^8^ | 0.992 |
| 4 | *Lactobacillus iners* | F：TCAAGTGACAGATGGCAACCGGAT  R：TTGCAATTATCTGTTGCCACAAC  P：FAM-TGCTTGAAATCACGTTCTGAA-MGB | CP045664.1 | F & R:0.5  P: 0.5 | 11.52 | 4.61×10^2^ to 4.61×10^8^ | 0.996 |
|  | *Lactobacillus jensenii* | F：GCCAGTTTACCTTCGTTAACCT  R：CGAGAACTAATTGCGTAAACACAG  P：ROX-TTCCCATAGTCCGTAGCAGT-MGB | CP046310.1 | F & R: 0.5  P: 0.25 | 11.55 | 4.62×10^2^ to 4.62×10^8^ | 0.995 |
|  | *Lactobacillus gasseri* | F：TCATTAATGTATGGCGTTCAAAGA  R：CCATCCGTTTATTGGGCAAGAG  P：CY5-TCACGCTATGAATCTTTTTCGCTTACGGCA-MGB | CP044412.1 | F & R: 0.5  P: 0.25 | 11.67 | 4.67×10^2^ to 4.67×10^8^ | 0.987 |
|  | *Lactobacillus crispatus* | F：GTGGGAATGTGTAAGTTTAGCAAAGTG  R：GTGGTTAATTGAATTGGCGCATAAGT  P：VIC-ACGGTTATCGTGAATAGAAAATTACCGATCC-BHQ1 | CP047142.1 | F & R: 0.5  P: 0.25 | 11.67 | 4.67×10^2^ to 4.67×10^8^ | 0.982 |

^a^ F, R, P represents the forward primer, the reverse primer and the probe, respectively.
