# Supplementary material for: Vaginal microbiota molecular profiling and diagnostic performance of artificial intelligence-assisted multiplex PCR testing in women with bacterial vaginosis: a single-center experience
Source: Front Cell Infect Microbiol. 2024 Apr 5;14:1377225. doi: 10.3389/fcimb.2024.1377225 (PMC11026559; doi:10.3389/fcimb.2024.1377225)
Supplement: Supplementary file 2 [file Table_2.docx]

**Supplemental Table 2**.  Clinical and laboratory data of all subjects in this study.

| **ID^a^** | **Gender** | **Age** | **Sym^b^** | **Laboratory diagnosis^c^** | **LE** | **Sp** | **Tr** | **Bl** | **Donders’**  **score** | **FDen^d^** | **NS** | **PH** | **FDiv^e^** | **Dominant bacteria^f^** | **β-glu** | **H2O2** | **PD** | **LG^g^** | **LEase** | **Cl** | **Sialidase** | **NAG** | **Clue cells**  **(counts)** | **Whiff test** |
| --- | --- | --- | --- | --- | --- | --- | --- | --- | --- | --- | --- | --- | --- | --- | --- | --- | --- | --- | --- | --- | --- | --- | --- | --- |
| T1 | Female | 44 | 1 | BV | <10 | - | - | - | 2 | III | 8 | 4.6 | II | Gram-Negative Bacilli | - | + | - | III | - | Ⅲ | - | + | 0-3 | + |
| T2 | Female | 42 | 1 | BV | <10 | - | - | - | 2 | III | 8 | 4.6 | II | Gram-Negative Bacilli | - | + | - | III | - | Ⅲ | - | - | 0-3 | + |
| T3 | Female | 41 | 1 | BV | <10 | - | - | - | 2 | III | 7 | 4.8 | II | Gram-Negative Bacilli | - | + | - | III | - | Ⅲ | - | - | 0-3 | + |
| T4 | Female | 29 | 1 | BV | <10 | - | - | - | 2 | III | 8 | 4.6 | II | Gram-Negative Bacilli | - | + | - | III | + | Ⅲ | ± | - | 0-3 | + |
| T5 | Female | 34 | 1 | BV | <10 | - | - | - | 2 | III | 8 | 4.8 | II | Gram-Negative Bacilli | - | + | - | III | ± | Ⅲ | - | ± | 0-3 | + |
| T6 | Female | 24 | 1 | BV | <10 | - | - | - | 2 | III | 7 | 4.4 | II | Gram-Negative Bacilli | - | + | - | III | ± | Ⅲ | - | - | 0-2 | + |
| T7 | Female | 31 | 1 | BV | <10 | - | - | - | 2 | III | 8 | 4.6 | II | Gram-Negative Bacilli | - | + | - | III | - | Ⅲ | - | - | 0-3 | + |
| T8 | Female | 31 | 1 | BV | <10 | - | - | - | 2 | III | 7 | 4.4 | II | Gram-Negative Bacilli | - | + | - | III | - | Ⅲ | - | ± | 0-2 | + |
| T9 | Female | 42 | 1 | BV | <10 | - | - | - | 2 | III | 7 | 4.6 | II | Gram-Negative Bacilli | - | + | - | III | ± | Ⅲ | - | ± | 0-3 | + |
| T10 | Female | 39 | 1 | BV | <10 | - | - | - | 2 | III | 7 | 4.6 | II | Gram-Negative Bacilli | - | + | - | III | - | Ⅲ | - | - | 0-2 | + |
| T11 | Female | 36 | 1 | BV | <10 | - | - | - | 2 | III | 8 | 4.6 | II | Gram-Negative Bacilli | - | + | - | III | - | Ⅲ | - | - | 0-3 | + |
| T12 | Female | 19 | 1 | BV | <10 | - | - | - | 2 | III | 8 | 4.6 | II | Gram-Negative Bacilli | - | + | - | III | - | Ⅲ | - | - | 0-3 | + |
| T13 | Female | 26 | 1 | BV | <10 | - | - | - | 2 | III | 7 | 4.6 | II | Gram-Negative Bacilli | - | + | - | III | - | Ⅲ | - | - | 0-3 | + |
| T14 | Female | 48 | 1 | BV | <10 | - | - | - | 2 | IV | 8 | 4.6 | II | Gram-Negative Bacilli | - | + | - | III | ± | Ⅲ | + | - | 0-3 | + |
| T15 | Female | 42 | 1 | BV | <10 | - | - | - | 2 | III | 8 | 4.8 | II | Gram-Negative Bacilli | - | + | - | III | - | Ⅲ | - | - | 0-2 | + |
| T16 | Female | 29 | 1 | BV | <10 | - | - | - | 2 | III | 7 | 4.6 | II | Gram-Negative Bacilli | - | + | - | III | - | Ⅲ | - | - | 0-2 | + |
| T17 | Female | 36 | 1 | BV | <10 | - | - | - | 2 | III | 7 | 4.6 | II | Gram-Negative Bacilli | - | + | - | III | - | Ⅲ | - | - | 0-3 | + |
| T18 | Female | 42 | 1 | BV | <10 | - | - | - | 2 | III | 8 | 4.8 | II | Gram-Negative Bacilli | - | + | - | III | - | Ⅲ | - | - | 0-3 | + |
| T19 | Female | 45 | 1 | BV | <10 | - | - | - | 2 | III | 8 | 4.6 | II | Gram-Negative Bacilli | - | + | - | III | + | Ⅲ | ± | - | 0-3 | + |
| T20 | Female | 23 | 1 | BV | <10 | - | - | - | 2 | III | 7 | 4.6 | II | Gram-Negative Bacilli | - | + | - | III | ± | Ⅲ | - | - | 0-3 | + |
| T21 | Female | 44 | 1 | BV | <10 | - | - | - | 2 | III | 8 | 4.6 | II | Gram-Negative Bacilli | - | + | - | III | - | Ⅲ | - | - | 0-2 | + |
| T22 | Female | 52 | 1 | BV | <10 | - | - | - | 2 | III | 8 | 4.6 | II | Gram-Negative Bacilli | - | + | - | III | + | Ⅲ | - | + | 0-3 | + |
| T23 | Female | 36 | 1 | BV | <10 | - | - | - | 1 | III | 7 | 4.6 | III | Gram-Negative Bacilli | - | + | - | IIb | ± | Ⅲ | ± | - | 0-3 | + |
| T24 | Female | 44 | 1 | BV | <10 | - | - | - | 2 | III | 7 | 4.6 | II | Gram-Negative Bacilli | - | + | - | III | - | Ⅲ | - | - | 0-2 | + |
| T25 | Female | 37 | 1 | BV | <10 | - | - | - | 2 | III | 8 | 4.6 | II | Gram-Negative Bacilli | - | + | - | III | ± | II | - | - | 0-3 | + |
| T26 | Female | 33 | 1 | BV | <10 | - | - | - | 2 | III | 8 | 4.6 | II | Gram-Negative Bacilli | - | + | - | III | - | Ⅲ | - | + | 0-3 | + |
| T27 | Female | 27 | 1 | BV | <10 | - | - | - | 2 | III | 8 | 4.6 | II | Gram-Negative Bacilli | - | + | - | III | - | Ⅲ | - | - | 0-3 | + |
| T28 | Female | 33 | 1 | BV | <10 | - | - | - | 2 | III | 8 | 4.8 | II | Gram-Negative Bacilli | - | + | - | III | - | Ⅲ | - | + | 0-3 | + |
| T29 | Female | 28 | 1 | BV | >10 | - | - | - | 2 | III | 8 | 4.6 | II | Gram-Negative Bacilli | - | + | - | III | - | Ⅲ | - | - | 0-2 | + |
| T30 | Female | 24 | 1 | BV | <10 | - | - | - | 2 | III | 8 | 4.6 | II | Gram-Negative Bacilli | - | + | - | III | - | Ⅲ | - | - | 0-2 | + |
| T31 | Female | 55 | 1 | BV | <10 | - | - | - | 2 | III | 8 | 4.6 | II | Gram-Negative Bacilli | - | + | - | III | ± | Ⅲ | - | - | 0-2 | + |
| T32 | Female | 32 | 1 | BV | <10 | - | - | - | 2 | III | 8 | 4.6 | II | Gram-Negative Bacilli | - | + | - | III | + | Ⅲ | ± | + | 0-3 | + |
| T33 | Female | 40 | 1 | BV | <10 | - | - | - | 2 | III | 7 | 4.6 | II | Gram-Negative Bacilli | - | + | - | III | + | Ⅲ | ± | + | 0-2 | + |
| T34 | Female | 23 | 1 | BV | <10 | - | - | - | 2 | III | 8 | 4.6 | II | Gram-Negative Bacilli | - | + | - | III | + | Ⅲ | ± | ± | 0-3 | + |
| T35 | Female | 31 | 1 | BV | <10 | - | - | - | 2 | III | 8 | 4.6 | II | Gram-Negative Bacilli | - | + | - | III | ± | Ⅲ | ± | + | 0-3 | + |
| T36 | Female | 24 | 1 | BV | <10 | - | - | - | 2 | III | 8 | 4.6 | II | Gram-Negative Bacilli | - | + | - | III | - | Ⅲ | - | - | 0-3 | + |
| T37 | Female | 38 | 1 | BV | <10 | - | - | - | 2 | III | 8 | 4.6 | II | Gram-Negative Bacilli | - | + | - | III | + | Ⅲ | + | - | 0-3 | + |
| T38 | Female | 46 | 1 | BV | <10 | - | - | - | 2 | III | 8 | 4.6 | II | Gram-Negative Bacilli | - | + | - | III | - | Ⅲ | - | - | 0-3 | + |
| T39 | Female | 33 | 1 | BV | <10 | - | - | - | 2 | III | 8 | 4.6 | II | Gram-Negative Bacilli | - | + | - | III | - | Ⅲ | - | - | 0-3 | + |
| T40 | Female | 37 | 1 | BV | <10 | - | - | - | 2 | IV | 8 | 4.6 | II | Gram-Negative Bacilli | - | + | - | III | + | Ⅲ | + | - | 0-3 | + |
| T41 | Female | 23 | 1 | BV | <10 | - | - | - | 2 | IV | 8 | 4.6 | II | Gram-Negative Bacilli | - | + | - | III | - | Ⅲ | - | + | 0-3 | + |
| T42 | Female | 31 | 1 | BV | <10 | - | - | - | 2 | IV | 8 | 4.6 | III | Gram-Negative Bacilli | - | + | - | III | + | Ⅲ | + | + | 0-3 | + |
| T43 | Female | 45 | 1 | BV | <10 | - | - | - | 2 | III | 8 | 4.6 | II | Gram-Negative Bacilli | - | + | - | III | ± | Ⅲ | - | - | 0-3 | + |
| T44 | Female | 54 | 1 | BV | <10 | - | - | - | 2 | III | 8 | 4.6 | II | Gram-Negative Bacilli | - | + | - | III | + | Ⅲ | ± | + | 0-3 | + |
| T45 | Female | 46 | 1 | BV | <10 | - | - | - | 2 | III | 8 | 4.6 | II | Gram-Negative Bacilli | - | + | - | III | - | Ⅲ | - | - | 0-3 | + |
| T46 | Female | 28 | 1 | BV | <10 | - | - | - | 2 | III | 8 | 4.6 | II | Gram-Negative Bacilli | - | + | - | III | - | Ⅲ | - | - | 0-5 | + |
| T47 | Female | 23 | 1 | BV | <10 | - | - | - | 2 | III | 7 | 4.8 | II | Gram-Negative Bacilli | - | + | - | III | - | Ⅲ | - | - | 0-3 | + |
| T48 | Female | 30 | 1 | BV | <10 | - | - | - | 2 | III | 7 | 4.6 | II | Gram-Negative Bacilli | - | + | - | III | ± | Ⅲ | ± | + | 0-3 | + |
| T49 | Female | 32 | 1 | BV | <10 | - | - | - | 2 | III | 8 | 4.6 | II | Gram-Negative Bacilli | - | + | - | III | - | Ⅲ | - | - | 0-3 | + |
| T50 | Female | 44 | 1 | BV | <10 | - | - | - | 2 | III | 8 | 4.8 | II | Gram-Negative Bacilli | - | + | - | III | - | Ⅲ | - | - | 0-3 | + |
| T51 | Female | 42 | 1 | BV | <10 | - | - | - | 2 | III | 8 | 4.6 | II | Gram-Negative Bacilli | - | + | - | III | ± | Ⅲ | - | + | 0-3 | + |
| T52 | Female | 22 | 1 | BV | <10 | - | - | - | 2 | III | 9 | 4.6 | II | Gram-Negative Bacilli | - | + | - | III | - | Ⅲ | - | - | 0-3 | + |
| T53 | Female | 32 | 1 | BV | <10 | - | - | - | 2 | III | 8 | 4.6 | II | Gram-Negative Bacilli | - | + | - | III | - | Ⅲ | - | - | 0-2 | + |
| T54 | Female | 53 | 1 | BV | <10 | - | - | - | 2 | III | 7 | 4.6 | II | Gram-Negative Bacilli | - | + | - | III | ± | Ⅲ | + | + | 0-2 | + |
| T55 | Female | 37 | 1 | BV | <10 | - | - | - | 2 | III | 8 | 4.4 | II | Gram-Negative Bacilli | - | + | - | III | ± | Ⅲ | ± | - | 0-2 | + |
| T56 | Female | 27 | 1 | BV | <10 | - | - | - | 2 | III | 8 | 4.6 | II | Gram-Negative Bacilli | - | + | - | III | + | Ⅲ | + | - | 0-3 | + |
| T57 | Female | 33 | 1 | BV | <10 | - | - | - | 2 | III | 7 | 4.6 | II | Gram-Negative Bacilli | - | + | - | III | - | Ⅲ | - | - | 0-3 | + |
| T58 | Female | 30 | 1 | BV | <10 | - | - | - | 2 | III | 7 | 4.8 | II | Gram-Negative Bacilli | - | + | - | III | + | Ⅲ | - | - | 0-3 | + |
| T59 | Female | 23 | 1 | BV | <10 | - | - | - | 2 | III | 8 | 4.6 | II | Gram-Negative Bacilli | - | + | - | III | ± | Ⅲ | - | - | 0-3 | + |
| T60 | Female | 35 | 1 | BV | <10 | - | - | - | 2 | III | 8 | 4.6 | II | Gram-Negative Bacilli | - | + | - | III | - | Ⅲ | - | - | 0-3 | + |
| T61 | Female | 46 | 1 | BV | <10 | - | - | - | 2 | III | 7 | 4.6 | II | Gram-Negative Bacilli | - | + | - | III | ± | Ⅲ | + | - | 0-3 | + |
| T62 | Female | 18 | 1 | BV | <10 | - | - | - | 2 | III | 8 | 4.8 | II | Gram-Negative Bacilli | - | + | - | III | + | Ⅲ | + | + | 0-3 | + |
| T63 | Female | 32 | 1 | BV | <10 | - | - | - | 2 | III | 8 | 4.6 | II | Gram-Negative Bacilli | - | + | - | III | - | Ⅲ | - | - | 0-3 | + |
| T64 | Female | 36 | 1 | BV | <10 | - | - | - | 2 | IV | 7 | 4.6 | II | Gram-Negative Bacilli | - | + | - | III | - | Ⅲ | - | - | 0-3 | + |
| T65 | Female | 21 | 1 | BV | <10 | - | - | - | 2 | III | 8 | 4.6 | II | Gram-Negative Bacilli | - | + | - | III | - | Ⅲ | - | - | 0-5 | + |
| T66 | Female | 29 | 1 | BV | <10 | - | - | - | 2 | III | 8 | 4.6 | II | Gram-Negative Bacilli | - | + | - | III | - | Ⅲ | - | - | 0-2 | + |
| T67 | Female | 25 | 1 | BV | <10 | - | - | - | 1 | III | 7 | 4.6 | II | Gram-Negative Bacilli | - | + | - | IIb | - | Ⅲ | - | - | 0-3 | + |
| T68 | Female | 32 | 1 | BV | >10 | - | - | - | 2 | III | 8 | 4.6 | II | Gram-Negative Bacilli | - | + | - | III | + | Ⅲ | - | - | 0-3 | + |
| T69 | Female | 50 | 1 | BV | <10 | - | - | - | 2 | IV | 7 | 4.6 | II | Gram-Negative Bacilli | - | + | - | III | - | Ⅲ | - | - | 0-3 | + |
| T70 | Female | 26 | 1 | BV | <10 | - | - | - | 2 | III | 7 | 4.6 | II | Gram-Negative Bacilli | - | + | - | III | - | Ⅲ | - | + | 0-2 | + |
| T71 | Female | 18 | 1 | BV | <10 | - | - | - | 1 | III | 7 | 4.8 | II | Gram-Negative Bacilli | - | + | - | III | - | Ⅲ | - | - | 0-3 | + |
| T72 | Female | 48 | 1 | BV | <10 | - | - | - | 1 | III | 8 | 4.6 | II | Gram-Negative Bacilli | - | + | - | III | - | Ⅲ | - | - | 0-3 | + |
| T73 | Female | 31 | 1 | BV | <10 | - | - | - | 2 | III | 8 | 4.6 | II | Gram-Negative Bacilli | - | + | - | III | - | Ⅲ | - | - | 0-3 | + |
| T74 | Female | 33 | 1 | BV | <10 | - | - | - | 2 | III | 7 | 4.6 | II | Gram-Negative Bacilli | - | + | - | III | - | Ⅲ | - | - | 0-3 | + |
| V1 | Female | 49 | 1 | BV | <10 | - | - | - | 2 | III | 8 | 4.6 | II | Gram-Negative Bacilli | - | + | - | III | + | Ⅲ | - | ± | 0-3 | + |
| V2 | Female | 35 | 1 | BV | <10 | - | - | - | 2 | III | 8 | 4.6 | II | Gram-Negative Bacilli | - | + | - | III | - | Ⅲ | - | - | 0-2 | + |
| V3 | Female | 39 | 1 | BV | <10 | - | - | - | 2 | III | 7 | 4.6 | II | Gram-Negative Bacilli | - | + | - | III | ± | Ⅲ | - | ± | 0-2 | + |
| V4 | Female | 56 | 1 | BV | <10 | - | - | - | 1 | III | 7 | 4.6 | II | Gram-Negative Bacilli | - | + | - | III | - | Ⅲ | - | - | 0-2 | + |
| V5 | Female | 24 | 1 | BV | <10 | - | - | - | 1 | III | 8 | 4.6 | II | Gram-Negative Bacilli | - | + | - | III | ± | Ⅲ | ± | ± | 0-3 | + |
| V6 | Female | 42 | 1 | BV | <10 | - | - | - | 2 | III | 8 | 4.6 | II | Gram-Negative Bacilli | - | + | - | III | ± | Ⅲ | - | - | 0-3 | + |
| V7 | Female | 36 | 1 | BV | <10 | - | - | - | 2 | III | 7 | 4.6 | II | Gram-Negative Bacilli | - | + | - | III | - | Ⅲ | - | ± | 0-2 | + |
| V8 | Female | 19 | 1 | BV | <10 | - | - | - | 2 | III | 8 | 4.6 | II | Gram-Negative Bacilli | - | + | - | III | + | Ⅱ | - | + | 0-3 | + |
| V9 | Female | 30 | 1 | BV | <10 | - | - | - | 2 | III | 7 | 4.6 | II | Gram-Negative Bacilli | - | + | - | III | + | Ⅲ | - | + | 0-3 | + |
| V10 | Female | 27 | 1 | BV | <10 | - | - | - | 2 | III | 7 | 4.4 | II | Gram-Negative Bacilli | - | + | - | III | ± | Ⅲ | + | + | 0-2 | + |
| V11 | Female | 23 | 1 | BV | <10 | - | - | - | 1 | III | 8 | 4.4 | II | Gram-Negative Bacilli | - | + | - | IIb | - | Ⅲ | - | - | 0-3 | + |
| V12 | Female | 30 | 1 | BV | <10 | - | - | - | 2 | III | 8 | 4.6 | II | Gram-Negative Bacilli | - | + | - | III | ± | Ⅲ | - | - | 0-3 | + |
| V13 | Female | 26 | 1 | BV | <10 | - | - | - | 2 | III | 8 | 4.4 | II | Gram-Negative Bacilli | - | + | - | III | - | Ⅲ | - | - | 0-2 | + |
| V14 | Female | 38 | 1 | BV | <10 | - | - | - | 1 | III | 9 | 4.6 | II | Gram-Negative Bacilli | - | + | - | III | ± | II | - | - | 0-2 | + |
| V15 | Female | 32 | 1 | BV | <10 | - | - | - | 1 | III | 7 | 4.6 | II | Gram-Negative Bacilli | - | + | - | III | - | Ⅲ | - | - | 2-5 | + |
| V16 | Female | 32 | 1 | BV | <10 | - | - | - | 2 | III | 8 | 4.6 | II | Gram-Negative Bacilli | - | + | - | III | + | II | - | - | 0-3 | + |
| V17 | Female | 27 | 1 | BV | <10 | - | - | - | 2 | III | 8 | 4.6 | II | Gram-Negative Bacilli | - | + | - | III | - | Ⅲ | - | - | 0-3 | + |
| V18 | Female | 28 | 1 | BV | <10 | - | - | - | 2 | III | 8 | 4.4 | II | Gram-Negative Bacilli | - | + | - | III | ± | Ⅲ | - | - | 0-3 | + |
| V19 | Female | 48 | 1 | BV | <10 | - | - | - | 2 | III | 8 | 4.6 | II | Gram-Negative Bacilli | - | + | - | III | + | Ⅲ | - | - | 0-3 | + |
| V20 | Female | 48 | 1 | BV | <10 | - | - | - | 2 | III | 7 | 4.6 | II | Gram-Negative Bacilli | - | + | - | III | + | Ⅲ | - | - | 0-2 | + |
| V21 | Female | 34 | 1 | BV | <10 | - | - | - | 2 | III | 7 | 4.6 | II | Gram-Negative Bacilli | - | + | - | III | + | Ⅲ | ± | ± | 2-5 | + |
| V22 | Female | 28 | 1 | BV | <10 | - | - | - | 2 | III | 8 | 4.6 | II | Gram-Negative Bacilli | - | + | - | III | + | Ⅲ | - | + | 0-3 | + |
| V23 | Female | 24 | 1 | BV | <10 | - | - | - | 2 | III | 8 | 4.6 | II | Gram-Negative Bacilli | - | + | - | III | ± | Ⅲ | - | - | 0-3 | + |
| V24 | Female | 47 | 1 | BV | <10 | - | - | - | 2 | III | 7 | 4.6 | II | Gram-Negative Bacilli | - | + | - | III | ++ | Ⅲ | - | - | 0-3 | + |
| V25 | Female | 39 | 1 | BV | <10 | - | - | - | 1 | III | 8 | 4.6 | II | Gram-Negative Bacilli | - | + | - | IIb | ++ | Ⅲ | ± | ± | 0-3 | + |
| V26 | Female | 46 | 1 | BV | <10 | - | - | - | 2 | III | 8 | 4.6 | II | Gram-Negative Bacilli | - | + | - | III | - | Ⅲ | - | - | 0-3 | + |
| V27 | Female | 38 | 1 | BV | <10 | - | - | - | 1 | III | 7 | 4.6 | II | Gram-Negative Bacilli | - | + | - | III | ± | Ⅱ | - | - | 0-3 | + |
| V28 | Female | 38 | 1 | BV | <10 | - | - | - | 2 | III | 7 | 4.8 | II | Gram-Negative Bacilli | - | + | - | III | - | Ⅲ | - | - | 0-2 | + |
| V29 | Female | 32 | 1 | BV | <10 | - | - | - | 2 | III | 8 | 4.6 | II | Gram-Negative Bacilli | - | + | - | III | - | Ⅲ | - | - | 0-3 | + |
| V30 | Female | 50 | 1 | BV | <10 | - | - | - | 2 | III | 8 | 4.6 | II | Gram-Negative Bacilli | - | + | - | III | ± | Ⅲ | - | - | 0-2 | + |
| V31 | Female | 24 | 1 | BV | <10 | - | - | - | 2 | III | 8 | 4.4 | II | Gram-Negative Bacilli | - | + | - | III | + | Ⅲ | - | - | 0-2 | + |
| V32 | Female | 43 | 1 | BV | <10 | - | - | - | 2 | III | 8 | 4.6 | II | Gram-Negative Bacilli | - | + | - | III | + | Ⅲ | - | + | 0-2 | + |
| V33 | Female | 43 | 1 | BV | <10 | - | - | - | 2 | III | 8 | 4.6 | II | Gram-Negative Bacilli | - | + | - | III | - | Ⅲ | - | - | 0-2 | + |
| V34 | Female | 38 | 1 | BV | <10 | - | - | - | 1 | III | 7 | 4.6 | III | Gram-Negative Bacilli | - | + | - | III | ± | Ⅲ | - | + | 0-2 | + |
| V35 | Female | 26 | 1 | BV | <10 | - | - | - | 2 | III | 7 | 4.6 | II | Gram-Negative Bacilli | - | + | - | III | - | Ⅲ | - | - | 0-2 | + |
| V36 | Female | 32 | 1 | BV | <10 | - | - | - | 2 | III | 8 | 4.6 | II | Gram-Negative Bacilli | - | + | - | III | - | Ⅲ | - | - | 0-2 | + |
| V37 | Female | 38 | 1 | BV | <10 | - | - | - | 2 | III | 8 | 4.6 | II | Gram-Negative Bacilli | - | + | - | III | - | Ⅲ | - | - | 0-3 | + |
| V38 | Female | 36 | 1 | BV | <10 | - | - | - | 1 | III | 8 | 4.6 | II | Gram-Negative Bacilli | - | + | - | III | ++ | Ⅱ | - | + | 0-2 | + |
| V39 | Female | 24 | 1 | BV | >10 | - | - | - | 2 | III | 7 | 4.6 | II | Gram-Negative Bacilli | - | + | - | III | + | Ⅲ | - | ± | 0-3 | + |
| V40 | Female | 35 | 1 | BV | <10 | - | - | - | 1 | III | 7 | 4.6 | II | Gram-Negative Bacilli | - | + | - | III | - | Ⅲ | - | - | 0-2 | + |
| V41 | Female | 31 | 1 | BV | <10 | - | - | - | 2 | III | 7 | 4.6 | II | Gram-Negative Bacilli | - | + | - | III | ± | Ⅲ | ± | + | 0-3 | + |
| V42 | Female | 27 | 1 | BV | <10 | - | - | - | 2 | III | 8 | 4.8 | II | Gram-Negative Bacilli | - | + | - | III | - | Ⅲ | - | - | 0-2 | + |
| V43 | Female | 36 | 1 | BV | <10 | - | - | - | 2 | III | 8 | 4.8 | II | Gram-Negative Bacilli | - | + | - | III | - | Ⅲ | - | - | 0-5 | + |
| V44 | Female | 37 | 1 | BV | <10 | - | - | - | 1 | III | 7 | 4.6 | II | Gram-Negative Bacilli | - | + | - | III | - | Ⅲ | - | - | 0-3 | + |
| V45 | Female | 33 | 1 | BV | <10 | - | - | - | 2 | III | 8 | 4.6 | II | Gram-Negative Bacilli | - | + | - | III | - | Ⅲ | - | - | 0-2 | + |
| V46 | Female | 21 | 1 | BV | <10 | - | - | - | 2 | III | 8 | 4.6 | II | Gram-Negative Bacilli | - | + | - | III | - | Ⅲ | - | - | 0-2 | + |
| V47 | Female | 38 | 1 | BV | <10 | - | - | - | 2 | III | 7 | 4.6 | II | Gram-Negative Bacilli | - | + | - | III | - | Ⅲ | - | - | 0-3 | + |
| V48 | Female | 34 | 1 | BV | <10 | - | - | - | 2 | III | 7 | 4.8 | II | Gram-Negative Bacilli | - | + | - | III | - | Ⅲ | - | - | 0-3 | + |
| V49 | Female | 22 | 1 | BV | <10 | - | - | - | 2 | IV | 8 | 4.6 | II | Gram-Negative Bacilli | - | + | - | III | + | Ⅲ | - | + | 0-3 | + |
| V50 | Female | 34 | 1 | BV | <10 | - | - | - | 2 | III | 8 | 4.6 | II | Gram-Negative Bacilli | - | + | - | III | - | Ⅲ | - | - | 0-2 | + |
| V51 | Female | 45 | 1 | BV | <10 | - | - | - | 2 | III | 7 | 4.4 | II | Gram-Negative Bacilli | - | + | - | III | - | Ⅲ | - | - | 0-2 | + |
| V52 | Female | 40 | 1 | BV | <10 | - | - | - | 1 | III | 8 | 4.6 | II | Gram-Negative Bacilli | - | + | - | III | ± | Ⅲ | - | - | 0-3 | + |
| V53 | Female | 42 | 1 | BV | <10 | - | - | - | 2 | III | 7 | 4.6 | II | Gram-Negative Bacilli | - | + | - | III | - | Ⅲ | - | - | 0-3 | + |
| V54 | Female | 35 | 1 | BV | <10 | - | - | - | 2 | III | 7 | 4.8 | II | Gram-Negative Bacilli | - | + | - | III | - | Ⅲ | - | - | 0-2 | + |
| V55 | Female | 34 | 1 | BV | <10 | - | - | - | 1 | III | 7 | 4.6 | II | Gram-Negative Bacilli | - | + | - | III | ± | Ⅲ | - | - | 0-3 | + |
| V56 | Female | 41 | 1 | BV | <10 | - | - | - | 1 | III | 8 | 4.6 | II | Gram-Negative Bacilli | - | + | - | III | - | Ⅲ | - | - | 0-3 | + |
| V57 | Female | 32 | 1 | BV | <10 | - | - | - | 2 | III | 8 | 4.6 | II | Gram-Negative Bacilli | - | + | - | III | + | Ⅲ | - | - | 0-3 | + |
| V58 | Female | 41 | 1 | BV | <10 | - | - | - | 2 | III | 7 | 4.6 | II | Gram-Negative Bacilli | - | + | - | III | ++ | Ⅲ | - | + | 0-3 | + |
| V59 | Female | 42 | 1 | BV | <10 | - | - | - | 2 | III | 8 | 4.6 | II | Gram-Negative Bacilli | - | + | - | III | - | Ⅲ | - | - | 0-3 | + |
| V60 | Female | 26 | 1 | BV | <10 | - | - | - | 2 | III | 8 | 4.6 | II | Gram-Negative Bacilli | - | + | - | IIb | ± | Ⅲ | - | + | 0-3 | + |
| V61 | Female | 41 | 1 | BV | <10 | - | - | - | 2 | III | 8 | 4.6 | II | Gram-Negative Bacilli | - | + | - | III | - | Ⅲ | - | - | 0-3 | + |
| V62 | Female | 36 | 1 | BV | <10 | - | - | - | 2 | III | 7 | 4.6 | II | Gram-Negative Bacilli | - | + | - | III | ± | Ⅲ | + | - | 0-3 | + |
| V63 | Female | 25 | 1 | BV | <10 | - | - | - | 2 | III | 8 | 4.8 | II | Gram-Negative Bacilli | - | + | - | IIb | ++ | Ⅲ | ± | ± | 0-3 | + |
| V64 | Female | 26 | 1 | BV | <10 | - | - | - | 2 | III | 8 | 4.6 | II | Gram-Negative Bacilli | - | + | - | III | - | Ⅲ | - | - | 0-3 | + |
| V65 | Female | 26 | 1 | BV | <10 | - | - | - | 2 | III | 7 | 4.6 | II | Gram-Negative Bacilli | - | + | - | III | ± | Ⅱ | - | - | 0-2 | + |
| V66 | Female | 32 | 1 | BV | <10 | - | - | - | 2 | III | 8 | 4.6 | II | Gram-Negative Bacilli | - | + | - | III | - | Ⅲ | - | - | 0-3 | + |
| V67 | Female | 34 | 1 | BV | <10 | - | - | - | 2 | III | 7 | 4.6 | II | Gram-Negative Bacilli | - | + | - | III | - | Ⅲ | - | - | 0-3 | + |
| V68 | Female | 34 | 1 | BV | <10 | - | - | - | 2 | III | 7 | 4.6 | II | Gram-Negative Bacilli | - | + | - | III | - | Ⅲ | - | - | 0-3 | + |
| V69 | Female | 52 | 1 | BV | <10 | - | - | - | 2 | III | 8 | 4.6 | II | Gram-Negative Bacilli | - | + | - | III | - | Ⅲ | - | - | 0-2 | + |
| V70 | Female | 40 | 1 | BV | <10 | - | - | - | 2 | III | 7 | 4.8 | II | Gram-Negative Bacilli | - | + | - | III | - | Ⅲ | - | - | 0-3 | + |
| V71 | Female | 30 | 1 | BV | <10 | - | - | - | 2 | IV | 8 | 4.6 | II | Gram-Negative Bacilli | - | + | - | III | - | Ⅲ | - | - | 0-2 | + |
| V72 | Female | 33 | 1 | BV | <10 | - | - | - | 2 | III | 8 | 4.4 | II | Gram-Negative Bacilli | - | + | - | III | - | Ⅲ | - | - | 0-2 | + |
| V73 | Female | 38 | 1 | BV | <10 | - | - | - | 2 | III | 8 | 4.6 | II | Gram-Negative Bacilli | - | + | - | III | - | Ⅲ | - | - | 0-2 | + |
| V74 | Female | 25 | 1 | BV | <10 | - | - | - | 2 | III | 8 | 4.6 | II | Gram-Negative Bacilli | - | + | - | III | + | Ⅲ | ± | + | 0-2 | + |
| V75 | Female | 55 | 1 | BV | <10 | - | - | - | 2 | III | 8 | 4.6 | II | Gram-Negative Bacilli | - | + | - | III | - | Ⅲ | - | - | 0-2 | + |
| V76 | Female | 19 | 1 | BV | <10 | - | - | - | 2 | III | 7 | 4.8 | II | Gram-Negative Bacilli | - | + | - | III | + | Ⅲ | ± | + | 0-2 | + |
| T75 | Female | 32 | 0 | Normal Flora | <10 | - | - | - | 1 | II | 1 | 4.4 | II | Gram-Positive Bacilli | - | + | - | I | - | II | - | - | - | - |
| T76 | Female | 28 | 0 | Normal Flora | <10 | - | - | - | 0 | II | 2 | 4.1 | II | Gram-Positive Bacilli | - | + | - | I | + | II | - | - | - | - |
| T77 | Female | 43 | 0 | Normal Flora | <10 | - | - | - | 0 | II | 0 | 4.1 | II | Gram-Positive Bacilli | - | + | - | I | ± | II | - | + | - | - |
| T78 | Female | 38 | 0 | Normal Flora | <10 | - | - | - | 0 | II | 0 | 4.1 | II | Gram-Positive Bacilli | - | + | - | I | ± | II | - | - | - | - |
| T79 | Female | 34 | 0 | Normal Flora | <10 | - | - | - | 0 | II | 2 | 4.1 | II | Gram-Positive Bacilli | - | + | - | I | - | II | - | - | - | - |
| T80 | Female | 30 | 0 | Normal Flora | <10 | - | - | - | 0 | II | 0 | 4.1 | II | Gram-Positive Bacilli | - | + | - | I | - | II | - | - | - | - |
| T81 | Female | 37 | 0 | Normal Flora | <10 | - | - | - | 0 | II | 0 | 4.6 | II | Gram-Positive Bacilli | - | + | - | I | - | II | - | - | - | - |
| T82 | Female | 29 | 0 | Normal Flora | <10 | - | - | - | 0 | II | 0 | 4.4 | II | Gram-Positive Bacilli | - | + | - | IIa | ± | II | - | ± | - | - |
| T83 | Female | 39 | 0 | Normal Flora | <10 | - | - | - | 0 | II | 0 | 4.1 | II | Gram-Positive Bacilli | - | + | - | I | - | II | - | + | - | - |
| T84 | Female | 45 | 0 | Normal Flora | <10 | - | - | - | 0 | II | 1 | 4.1 | II | Gram-Positive Bacilli | - | + | - | I | ± | II | - | + | - | - |
| T85 | Female | 37 | 0 | Normal Flora | <10 | - | - | - | 0 | II | 0 | 4.4 | II | Gram-Positive Bacilli | - | + | - | I | + | II | - | - | - | - |
| T86 | Female | 26 | 0 | Normal Flora | <10 | - | - | - | 0 | II | 0 | 4.1 | II | Gram-Positive Bacilli | - | + | - | IIa | - | II | - | - | - | - |
| T87 | Female | 32 | 0 | Normal Flora | <10 | - | - | - | 0 | II | 0 | 4.1 | II | Gram-Positive Bacilli | - | + | - | IIa | - | II | - | - | - | - |
| T88 | Female | 23 | 0 | Normal Flora | <10 | - | - | - | 1 | II | 2 | 4.4 | II | Gram-Positive Bacilli | - | + | - | I | + | II | - | - | - | - |
| T89 | Female | 34 | 0 | Normal Flora | <10 | - | - | - | 1 | II | 1 | 4.4 | II | Gram-Positive Bacilli | - | + | - | I | - | II | - | - | - | - |
| T90 | Female | 41 | 0 | Normal Flora | <10 | - | - | - | 0 | II | 0 | 4.1 | II | Gram-Positive Bacilli | - | + | - | I | - | II | - | - | - | - |
| T91 | Female | 29 | 0 | Normal Flora | <10 | - | - | - | 1 | II | 0 | 4.1 | II | Gram-Positive Bacilli | - | + | - | I | - | II | - | + | - | - |
| T92 | Female | 27 | 0 | Normal Flora | <10 | - | - | - | 1 | II | 0 | 4.4 | II | Gram-Positive Bacilli | - | + | - | I | - | II | - | + | - | - |
| T93 | Female | 35 | 0 | Normal Flora | <10 | - | - | - | 0 | II | 0 | 4.1 | II | Gram-Positive Bacilli | - | + | - | I | + | II | - | ± | - | - |
| T94 | Female | 23 | 0 | Normal Flora | <10 | - | - | - | 0 | II | 0 | 4.4 | II | Gram-Positive Bacilli | - | + | - | IIa | ± | II | - | - | - | - |
| T95 | Female | 51 | 0 | Normal Flora | <10 | - | - | - | 0 | II | 0 | 4.1 | II | Gram-Positive Bacilli | - | + | - | I | - | II | - | ± | - | - |
| T96 | Female | 36 | 0 | Normal Flora | <10 | - | - | - | 0 | II | 0 | 4.1 | II | Gram-Positive Bacilli | - | + | - | I | + | II | - | - | - | - |
| T97 | Female | 30 | 0 | Normal Flora | <10 | - | - | - | 0 | II | 0 | 4.1 | II | Gram-Positive Bacilli | - | + | - | I | - | II | - | - | - | - |
| T98 | Female | 36 | 0 | Normal Flora | <10 | - | - | - | 0 | II | 1 | 4.4 | II | Gram-Positive Bacilli | - | + | - | I | - | II | - | - | - | - |
| T99 | Female | 27 | 0 | Normal Flora | <10 | - | - | - | 0 | II | 0 | 4.1 | II | Gram-Positive Bacilli | - | + | - | I | - | II | - | - | - | - |
| V77 | Female | 28 | 0 | Normal Flora | <10 | - | - | - | 1 | II | 0 | 4.1 | II | Gram-Positive Bacilli | - | + | - | I | - | II | - | - | - | - |
| V78 | Female | 33 | 0 | Normal Flora | <10 | - | - | - | 1 | II | 0 | 4.1 | II | Gram-Positive Bacilli | - | + | - | I | - | II | - | - | - | - |
| V79 | Female | 32 | 0 | Normal Flora | <10 | - | - | - | 0 | II | 1 | 4.1 | II | Gram-Positive Bacilli | - | + | - | I | - | II | - | - | - | - |
| V80 | Female | 39 | 0 | Normal Flora | <10 | - | - | - | 1 | II | 1 | 4.1 | II | Gram-Positive Bacilli | - | + | - | I | - | II | - | - | - | - |
| V81 | Female | 39 | 0 | Normal Flora | <10 | - | - | - | 0 | II | 0 | 4.1 | II | Gram-Positive Bacilli | - | + | - | I | - | II | - | - | - | - |
| V82 | Female | 37 | 0 | Normal Flora | <10 | - | - | - | 0 | II | 0 | 4.4 | II | Gram-Positive Bacilli | - | + | - | I | - | II | - | - | - | - |
| V83 | Female | 29 | 0 | Normal Flora | <10 | - | - | - | 0 | II | 0 | 4.1 | II | Gram-Positive Bacilli | - | + | - | IIa | - | II | - | ± | - | - |
| V84 | Female | 31 | 0 | Normal Flora | <10 | - | - | - | 0 | II | 0 | 4.4 | II | Gram-Positive Bacilli | - | + | - | I | + | II | - | - | - | - |
| V85 | Female | 32 | 0 | Normal Flora | <10 | - | - | - | 0 | II | 0 | 4.1 | II | Gram-Positive Bacilli | - | + | - | I | ± | II | - | - | - | - |
| V86 | Female | 30 | 0 | Normal Flora | <10 | - | - | - | 0 | II | 0 | 4.1 | II | Gram-Positive Bacilli | - | + | - | I | - | II | - | + | - | - |
| V87 | Female | 30 | 0 | Normal Flora | <10 | - | - | - | 0 | II | 3 | 4.1 | II | Gram-Positive Bacilli | - | + | - | I | - | II | - | ± | - | - |
| V88 | Female | 36 | 0 | Normal Flora | <10 | - | - | - | 0 | II | 0 | 4.4 | II | Gram-Positive Bacilli | - | + | - | IIa | ± | II | - | - | - | - |
| V89 | Female | 33 | 0 | Normal Flora | <10 | - | - | - | 0 | II | 0 | 4.1 | II | Gram-Positive Bacilli | - | + | - | I | - | Ⅲ | - | - | - | - |
| V90 | Female | 26 | 0 | Normal Flora | <10 | - | - | - | 0 | II | 0 | 4.1 | II | Gram-Positive Bacilli | - | ± | - | IIa | - | II | - | - | - | - |
| V91 | Female | 26 | 0 | Normal Flora | <10 | - | - | - | 0 | II | 0 | 4.1 | II | Gram-Positive Bacilli | - | + | - | I | - | II | - | - | - | - |
| V92 | Female | 29 | 0 | Normal Flora | <10 | - | - | - | 0 | II | 0 | 4.1 | II | Gram-Positive Bacilli | - | + | - | IIa | - | II | - | - | - | - |
| V93 | Female | 27 | 0 | Normal Flora | <10 | - | - | - | 0 | II | 0 | 4.4 | II | Gram-Positive Bacilli | - | + | - | I | - | II | - | - | - | - |
| V94 | Female | 32 | 0 | Normal Flora | <10 | - | - | - | 2 | II | 0 | 4.4 | II | Gram-Positive Bacilli | - | + | - | IIa | - | II | - | + | - | - |
| V95 | Female | 38 | 0 | Normal Flora | <10 | - | - | - | 0 | II | 0 | 4.4 | II | Gram-Positive Bacilli | - | + | - | I | + | II | - | - | - | - |
| V96 | Female | 32 | 0 | Normal Flora | <10 | - | - | - | 1 | II | 0 | 4.4 | II | Gram-Positive Bacilli | - | + | - | I | - | II | - | - | - | - |
| V97 | Female | 18 | 0 | Normal Flora | <10 | - | - | - | 1 | II | 0 | 4.1 | II | Gram-Positive Bacilli | - | + | - | I | - | II | - | - | - | - |
| V98 | Female | 40 | 0 | Normal Flora | <10 | - | - | - | 0 | II | 0 | 4.1 | II | Gram-Positive Bacilli | - | + | - | IIa | ± | II | - | + | - | - |
| V99 | Female | 34 | 0 | Normal Flora | <10 | - | - | - | 0 | II | 1 | 4.1 | II | Gram-Positive Bacilli | - | + | - | I | - | II | - | - | - | - |
| V100 | Female | 30 | 0 | Normal Flora | <10 | - | - | - | 1 | II | 0 | 4.1 | II | Gram-Positive Bacilli | - | + | - | I | - | II | - | - | - | - |
| V101 | Female | 38 | 0 | Normal Flora | <10 | - | - | - | 0 | II | 0 | 4.1 | II | Gram-Positive Bacilli | - | + | - | I | - | II | - | - | - | - |
| V102 | Female | 29 | 0 | Normal Flora | <10 | - | - | - | 0 | II | 0 | 4.1 | II | Gram-Positive Bacilli | - | + | - | IIa | - | II | - | - | - | - |
| V103 | Female | 24 | 0 | Normal Flora | <10 | - | - | - | 1 | II | 0 | 4.4 | II | Gram-Positive Bacilli | - | + | - | I | ± | II | - | - | - | - |
| V104 | Female | 33 | 0 | Normal Flora | <10 | - | - | - | 1 | II | 1 | 4.4 | II | Gram-Positive Bacilli | - | + | - | I | ± | II | - | - | - | - |
| V105 | Female | 37 | 0 | Normal Flora | <10 | - | - | - | 0 | II | 0 | 4.1 | II | Gram-Positive Bacilli | - | + | - | I | - | II | - | - | - | - |
| V106 | Female | 35 | 0 | Normal Flora | <10 | - | - | - | 0 | II | 2 | 4.4 | II | Gram-Positive Bacilli | - | + | - | I | - | II | - | - | - | - |
| V107 | Female | 30 | 0 | Normal Flora | <10 | - | - | - | 2 | II | 0 | 4.1 | II | Gram-Positive Bacilli | - | + | - | I | - | II | - | - | - | - |
| V108 | Female | 31 | 0 | Normal Flora | <10 | - | - | - | 1 | II | 2 | 4.1 | II | Gram-Positive Bacilli | - | + | - | IIa | - | II | - | - | - | - |
| V109 | Female | 25 | 0 | Normal Flora | <10 | - | - | - | 0 | II | 0 | 4.4 | II | Gram-Positive Bacilli | - | + | - | I | - | II | - | - | - | - |
| V110 | Female | 45 | 0 | Normal Flora | <10 | - | - | - | 1 | II | 2 | 4.1 | II | Gram-Positive Bacilli | - | + | - | I | - | II | - | ± | - | - |
| V111 | Female | 36 | 0 | Normal Flora | <10 | - | - | - | 0 | II | 0 | 4.1 | II | Gram-Positive Bacilli | - | + | - | I | + | II | - | - | - | - |
| V112 | Female | 29 | 0 | Normal Flora | <10 | - | - | - | 0 | II | 0 | 4.1 | II | Gram-Positive Bacilli | - | + | - | IIa | - | II | - | + | - | - |
| V113 | Female | 32 | 0 | Normal Flora | <10 | - | - | - | 0 | II | 0 | 4.1 | II | Gram-Positive Bacilli | - | + | - | I | ± | II | - | - | - | - |
| V114 | Female | 40 | 0 | Normal Flora | <10 | - | - | - | 0 | II | 1 | 4.1 | II | Gram-Positive Bacilli | - | + | - | I | - | II | - | - | - | - |
| V115 | Female | 27 | 0 | Normal Flora | <10 | - | - | - | 1 | II | 0 | 4.4 | II | Gram-Positive Bacilli | - | + | - | I | - | II | - | - | - | - |
| V116 | Female | 30 | 0 | Normal Flora | <10 | - | - | - | 0 | II | 0 | 4.4 | II | Gram-Positive Bacilli | - | + | - | I | - | II | - | - | - | - |
| V117 | Female | 51 | 0 | Normal Flora | <10 | - | - | - | 1 | II | 0 | 4.1 | II | Gram-Positive Bacilli | - | + | - | IIa | - | II | - | - | - | - |
| V118 | Female | 29 | 0 | Normal Flora | <10 | - | - | - | 0 | II | 2 | 4.1 | II | Gram-Positive Bacilli | - | + | - | IIa | - | II | - | + | - | - |
| V119 | Female | 29 | 0 | Normal Flora | <10 | - | - | - | 1 | II | 0 | 4.4 | II | Gram-Positive Bacilli | - | + | - | IIa | ± | II | - | + | - | - |
| V120 | Female | 27 | 0 | Normal Flora | <10 | - | - | - | 1 | II | 0 | 4.1 | II | Gram-Positive Bacilli | - | + | - | IIa | - | II | - | - | - | - |
| V121 | Female | 23 | 0 | Normal Flora | <10 | - | - | - | 0 | II | 0 | 4.1 | II | Gram-Positive Bacilli | - | + | - | IIa | - | II | - | - | - | - |
| V122 | Female | 28 | 0 | Normal Flora | <10 | - | - | - | 0 | II | 0 | 4.1 | II | Gram-Positive Bacilli | - | + | - | IIa | - | II | - | + | - | - |
| V123 | Female | 28 | 0 | Normal Flora | <10 | - | - | - | 0 | II | 0 | 4.4 | II | Gram-Positive Bacilli | - | + | - | I | ± | II | - | - | - | - |
| V124 | Female | 25 | 0 | Normal Flora | <10 | - | - | - | 0 | II | 1 | 4.1 | II | Gram-Positive Bacilli | - | + | - | I | - | II | - | - | - | - |
| V125 | Female | 33 | 0 | Normal Flora | <10 | - | - | - | 0 | II | 0 | 4.1 | II | Gram-Positive Bacilli | - | + | - | I | - | II | - | - | - | - |
| V126 | Female | 32 | 0 | Normal Flora | <10 | - | - | - | 0 | II | 0 | 4.1 | II | Gram-Positive Bacilli | - | + | - | I | ± | II | - | - | - | - |
| V127 | Female | 37 | 0 | Normal Flora | <10 | - | - | - | 1 | II | 0 | 4.1 | II | Gram-Positive Bacilli | - | + | - | I | - | II | - | - | - | - |
| V128 | Female | 23 | 0 | Normal Flora | <10 | - | - | - | 0 | II | 1 | 4.1 | II | Gram-Positive Bacilli | - | + | - | I | - | II | - | ± | - | - |
| V129 | Female | 28 | 0 | Normal Flora | <10 | - | - | - | 0 | II | 0 | 4.1 | II | Gram-Positive Bacilli | - | + | - | I | - | II | - | - | - | - |
| V130 | Female | 28 | 0 | Normal Flora | <10 | - | - | - | 0 | II | 0 | 4.1 | II | Gram-Positive Bacilli | - | + | - | I | ± | II | - | ± | - | - |
| V131 | Female | 25 | 0 | Normal Flora | <10 | - | - | - | 0 | II | 0 | 4.1 | II | Gram-Positive Bacilli | - | + | - | I | - | II | - | - | - | - |
| V132 | Female | 33 | 0 | Normal Flora | <10 | - | - | - | 1 | II | 0 | 4.1 | II | Gram-Positive Bacilli | - | + | - | I | - | II | - | - | - | - |
| V133 | Female | 32 | 0 | Normal Flora | <10 | - | - | - | 1 | II | 2 | 4.1 | II | Gram-Positive Bacilli | - | + | - | I | ± | II | - | - | - | - |
| V134 | Female | 37 | 0 | Normal Flora | <10 | - | - | - | 0 | III | 1 | 4.1 | II | Gram-Positive Bacilli | - | + | - | I | ± | II | - | + | - | - |
| V135 | Female | 40 | 0 | Normal Flora | <10 | - | - | - | 1 | II | 2 | 4.4 | II | Gram-Positive Bacilli | - | + | - | IIa | + | II | - | - | - | - |
| V136 | Female | 29 | 0 | Normal Flora | <10 | - | - | - | 0 | II | 1 | 4.4 | II | Gram-Positive Bacilli | - | + | - | I | - | II | - | - | - | - |
| V137 | Female | 25 | 0 | Normal Flora | <10 | - | - | - | 0 | II | 1 | 4.1 | II | Gram-Positive Bacilli | - | + | - | I | - | II | - | - | - | - |
| V138 | Female | 29 | 0 | Normal Flora | <10 | - | - | - | 0 | II | 1 | 4.1 | II | Gram-Positive Bacilli | - | + | - | IIa | ++ | II | - | - | - | - |
| V139 | Female | 31 | 0 | Normal Flora | <10 | - | - | - | 0 | II | 0 | 4.1 | II | Gram-Positive Bacilli | - | + | - | I | - | II | - | ± | - | - |
| V140 | Female | 32 | 0 | Normal Flora | <10 | - | - | - | 0 | II | 0 | 4.1 | II | Gram-Positive Bacilli | - | + | - | I | - | II | - | - | - | - |
| V141 | Female | 28 | 0 | Normal Flora | <10 | - | - | - | 1 | II | 0 | 4.1 | I | Gram-Positive Bacilli | - | + | - | I | - | II | - | - | - | - |
| V142 | Female | 33 | 0 | Normal Flora | <10 | - | - | - | 0 | II | 0 | 4.1 | II | Gram-Positive Bacilli | - | + | - | I | - | II | - | - | - | - |
| V143 | Female | 27 | 0 | Normal Flora | <10 | - | - | - | 1 | II | 0 | 4.1 | II | Gram-Positive Bacilli | - | + | - | I | - | II | - | - | - | - |
| V144 | Female | 34 | 0 | Normal Flora | <10 | - | - | - | 0 | II | 0 | 4.1 | II | Gram-Positive Bacilli | - | + | - | I | ± | II | - | - | - | - |
| V145 | Female | 28 | 0 | Normal Flora | <10 | - | - | - | 0 | II | 0 | 4.4 | II | Gram-Positive Bacilli | - | + | - | IIa | ± | II | - | + | - | - |
| V146 | Female | 25 | 0 | Normal Flora | <10 | - | - | - | 0 | II | 0 | 4.1 | II | Gram-Positive Bacilli | - | + | - | IIa | - | II | - | - | - | - |
| V147 | Female | 22 | 0 | Normal Flora | <10 | - | - | - | 1 | II | 0 | 4.1 | II | Gram-Positive Bacilli | - | + | - | I | - | II | - | + | - | - |
| V148 | Female | 30 | 0 | Normal Flora | <10 | - | - | - | 1 | II | 0 | 4.1 | II | Gram-Positive Bacilli | - | + | - | I | - | II | - | - | - | - |
| V149 | Female | 34 | 0 | Normal Flora | <10 | - | - | - | 1 | II | 0 | 4.4 | II | Gram-Positive Bacilli | - | + | - | I | - | II | - | - | - | - |
| V150 | Female | 31 | 0 | Normal Flora | <10 | - | - | - | 0 | II | 0 | 4.1 | II | Gram-Positive Bacilli | - | + | - | I | - | II | - | - | - | - |
| V151 | Female | 31 | 0 | Normal Flora | <10 | - | - | - | 0 | II | 1 | 4.4 | II | Gram-Positive Bacilli | - | + | - | I | - | II | - | + | - | - |
| V152 | Female | 28 | 0 | Normal Flora | <10 | - | - | - | 0 | II | 0 | 4.4 | II | Gram-Positive Bacilli | - | + | - | I | - | II | - | + | - | - |
| V153 | Female | 35 | 0 | Normal Flora | <10 | - | - | - | 0 | II | 0 | 4.1 | II | Gram-Positive Bacilli | - | ± | - | IIa | - | II | - | - | - | - |
| V154 | Female | 35 | 0 | Normal Flora | <10 | - | - | - | 2 | II | 1 | 4.4 | II | Gram-Positive Bacilli | - | + | - | I | - | II | - | - | - | - |
| V155 | Female | 26 | 0 | Normal Flora | <10 | - | - | - | 0 | II | 0 | 4.1 | II | Gram-Positive Bacilli | - | + | - | I | - | II | - | - | - | - |
| V156 | Female | 28 | 0 | Normal Flora | <10 | - | - | - | 0 | II | 0 | 4.1 | II | Gram-Positive Bacilli | - | + | - | I | - | II | - | - | - | - |
| V157 | Female | 37 | 0 | Normal Flora | <10 | - | - | - | 0 | II | 0 | 4.4 | II | Gram-Positive Bacilli | - | + | - | I | - | II | - | ± | - | - |
| V158 | Female | 27 | 0 | Normal Flora | <10 | - | - | - | 2 | II | 0 | 4.1 | II | Gram-Positive Bacilli | - | + | - | I | - | II | - | - | - | - |
| V159 | Female | 27 | 0 | Normal Flora | <10 | - | - | - | 0 | II | 0 | 4.4 | II | Gram-Positive Bacilli | - | + | - | I | - | II | - | - | - | - |
| M1 | Female | 26 | 1 | Intermediate Microbiota | <10 | - | - | - | 2 | II | 6 | 4.6 | II | Gram-Negative Bacilli | - | + | - | III | - | Ⅲ | - | - | - | - |
| M2 | Female | 37 | 1 | Intermediate Microbiota | <10 | - | - | - | 2 | II | 6 | 4.8 | II | Gram-Negative Bacilli | - | + | - | III | - | Ⅲ | - | - | - | - |
| M3 | Female | 25 | 1 | Intermediate Microbiota | <10 | - | - | - | 1 | III | 6 | 4.6 | II | Gram-Negative Bacilli | - | + | - | IIb | - | Ⅲ | - | - | - | - |
| M4 | Female | 35 | 1 | Intermediate Microbiota | <10 | - | - | - | 2 | II | 6 | 4.6 | II | Gram-Negative Bacilli | - | + | - | III | + | Ⅲ | - | - | - | - |
| M5 | Female | 44 | 1 | Intermediate Microbiota | <10 | - | - | - | 2 | II | 6 | 4.6 | II | Gram-Negative Bacilli | - | + | - | III | - | Ⅲ | - | - | - | - |
| M6 | Female | 30 | 1 | Intermediate Microbiota | <10 | - | - | - | 2 | II | 6 | 4.6 | II | Gram-Negative Bacilli | - | + | - | III | - | Ⅲ | - | + | 0-2 | + |
| M7 | Female | 31 | 1 | Intermediate Microbiota | >10 | - | - | - | 1 | III | 6 | 4.6 | II | Gram-Negative Bacilli | - | + | - | IIb | - | Ⅲ | - | - | - | + |
| M8 | Female | 27 | 1 | Intermediate Microbiota | <10 | - | - | - | 1 | III | 5 | 4.4 | II | Gram-Negative Bacilli | - | + | - | IIb | - | Ⅲ | - | - | 0-2 | + |
| M9 | Female | 33 | 1 | Intermediate Microbiota | <10 | - | - | - | 2 | III | 6 | 4.6 | II | Gram-Negative Bacilli | - | + | - | III | - | Ⅲ | - | - | - | - |
| M10 | Female | 46 | 1 | Intermediate Microbiota | <10 | - | - | - | 2 | II | 6 | 4.6 | II | Gram-Negative Bacilli | - | + | - | III | - | Ⅲ | - | - | 0-2 | + |
| M11 | Female | 46 | 1 | Intermediate Microbiota | >10 | - | - | - | 2 | III | 5 | 4.6 | II | Gram-Negative Bacilli | - | + | - | IIb | + | Ⅲ | - | ± | - | + |
| M12 | Female | 30 | 1 | Intermediate Microbiota | <10 | - | - | - | 2 | II | 6 | 4.6 | II | Gram-Negative Bacilli | - | + | - | III | - | Ⅲ | - | - | - | - |
| M13 | Female | 26 | 1 | Intermediate Microbiota | <10 | - | - | - | 2 | III | 6 | 4.6 | II | Gram-Negative Bacilli | - | + | - | III | ± | Ⅲ | - | ± | - | - |
| M14 | Female | 24 | 1 | Intermediate Microbiota | <10 | - | - | - | 1 | III | 5 | 4.6 | II | Gram-Negative Bacilli | - | + | - | IIb | - | Ⅲ | - | - | 0-3 | + |
| M15 | Female | 29 | 1 | Intermediate Microbiota | <10 | - | - | - | 1 | III | 6 | 4.6 | II | Gram-Negative Bacilli | - | + | - | IIb | ± | Ⅲ | ± | ± | 0-3 | + |
| M16 | Female | 42 | 1 | Intermediate Microbiota | >10 | - | - | - | 2 | III | 6 | 4.6 | II | Gram-Negative Bacilli | - | + | - | IIb | ± | Ⅲ | - | - | - | + |
| M17 | Female | 28 | 1 | Intermediate Microbiota | >10 | - | - | - | 2 | III | 5 | 4.6 | II | Gram-Negative Bacilli | - | ± | - | IIb | - | Ⅲ | - | ± | 0-2 | + |
| M18 | Female | 28 | 1 | Intermediate Microbiota | >10 | - | - | - | 2 | III | 6 | 4.6 | II | Gram-Negative Bacilli | - | + | - | IIb | + | Ⅱ | - | + | - | + |
| M19 | Female | 30 | 1 | Intermediate Microbiota | <10 | - | - | - | 2 | II | 6 | 4.6 | II | Gram-Negative Bacilli | - | + | - | III | + | Ⅲ | - | + | - | + |
| M20 | Female | 27 | 1 | Intermediate Microbiota | <10 | - | - | - | 2 | II | 6 | 4.6 | II | Gram-Negative Bacilli | - | + | - | III | ± | Ⅲ | + | + | - | + |
| M21 | Female | 28 | 1 | Intermediate Microbiota | <10 | - | - | - | 1 | III | 6 | 4.6 | II | Gram-Negative Bacilli | - | + | - | IIb | - | Ⅲ | - | - | - | - |
| M22 | Female | 24 | 1 | Intermediate Microbiota | >10 | - | - | - | 2 | III | 6 | 4.6 | II | Gram-Negative Bacilli | - | + | - | IIb | ± | Ⅲ | - | - | - | - |
| M23 | Female | 36 | 1 | Intermediate Microbiota | >10 | - | - | - | 2 | II | 5 | 4.4 | II | Gram-Negative Bacilli | - | + | - | IIb | - | Ⅲ | - | - | 0-2 | + |
| M24 | Female | 45 | 1 | Intermediate Microbiota | <10 | - | - | - | 1 | II | 5 | 4.6 | II | Gram-Negative Bacilli | - | + | - | IIb | ± | II | - | - | - | - |
| M25 | Female | 29 | 1 | Intermediate Microbiota | <10 | - | - | - | 1 | II | 6 | 4.6 | II | Gram-Negative Bacilli | - | + | - | IIb | - | Ⅲ | - | - | - | - |
| M26 | Female | 48 | 1 | Intermediate Microbiota | >10 | - | - | - | 2 | II | 5 | 4.8 | II | Gram-Negative Bacilli | - | + | - | IIb | + | II | - | - | - | - |
| M27 | Female | 53 | 1 | Intermediate Microbiota | <10 | - | - | - | 2 | II | 6 | 4.4 | II | Gram-Negative Bacilli | - | + | - | III | - | Ⅲ | - | - | - | + |
| M28 | Female | 40 | 1 | Intermediate Microbiota | >10 | - | - | - | 2 | II | 6 | 4.6 | II | Gram-Negative Bacilli | - | + | - | IIb | ± | Ⅲ | - | - | - | - |
| M29 | Female | 25 | 1 | Intermediate Microbiota | <10 | - | - | - | 2 | II | 6 | 4.6 | II | Gram-Negative Bacilli | - | + | - | III | + | Ⅲ | - | - | - | - |
| M30 | Female | 29 | 1 | Intermediate Microbiota | >10 | - | - | - | 2 | II | 5 | 4.6 | II | Gram-Negative Bacilli | - | + | - | IIb | + | Ⅲ | - | - | - | - |

The Gram stained smears were observed under the light microscope (Olympus) using the oil immersion objective (X100).

^a^T represents that the sample belongs to the modeling group; V and M represents that the sample belongs to the validation cohort.

^b^symptoms, 1 represents the patient's complaint of vaginal itching, pain, abnormal discharge, etc., and 0 represents the absence of vaginitis related symptoms.

^c^Normal flora: the density of vaginal flora was grade II-III, the diversity of vaginal flora was grade II-III, the dominant bacteria was Lactobacillus spp., no other pathogens were detected and the vaginal microbial function was normal.

^d^Flora density: Grade I, the average number of bacteria was 1-9/field; Grade II, the average number of bacteria was 10-99/field; Grade III, the average number of bacteria is more than 100/field; Grade IV, bacteria aggregated in clusters, or densely covered the mucosal epithelial cells.

^e^Flora diversity: Grade I, 1-3 species of bacteria; Grade II, 4-6 species of bacteria; Grade III, 7-9 species of bacteria; Grade IV, more than 10 bacterial species;

^f^Dominant bacteria: the bacteria with the largest biomass or population density in the flora.

^g^Lactobacillary Grades: Grade I, many lactobacilli, no other bacteria; Grade IIa, mixed flora but mainly Lactobacillus; Grade IIb, mixed flora, but the proportion of Lactobacillus was significantly lower than that of other bacteria; Grade III, severe reduction or absence of Lactobacillus and excessive growth of other bacteria.

Age, Age at diagnosis. Sym, symptoms. LE, leukocytes. Sp, spore. Tr, Trichomonas. Bl, blastospore. FDen, flora density. NS, Nugent scores. FDiv, flora diversity. *β*-glu, *β*-glucuronidase. H2O2, Hydrogen peroxide. PD, pseudohyphae. LG, Lactobacillary Grades. LEase, leukocyte esterase. Cl, cleanliness. NAG, N-acetylglucosaminidase.
